# Exploring experiential learning within interprofessional practice education initiatives for pre-licensure healthcare students: a scoping review

**DOI:** 10.1186/s12909-024-05114-w

**Published:** 2024-02-13

**Authors:** Daniel A. Nagel, Jamie L. Penner, Gayle Halas, Mark T. Philip, Carol A. Cooke

**Affiliations:** 1 College of Nursing, Rady Faculty of Health Sciences, University of Manitoba, Winnipeg, Canada; 2 Rady Chair in Interprofessional Collaborative Practice, Rady Faculty of Health Sciences, University of Manitoba, Winnipeg, Canada; 3 Neil John Maclean Health Sciences Library, University of Manitoba, Winnipeg, Canada

**Keywords:** Experiential learning, Health professions education, Healthcare education, Interprofessional practice, Interprofessional collaboration, Interprofessional education, Pre-licensure students, Scoping review, Service learning, Systematic review

## Abstract

**Background:**

Interprofessional collaborative team-based approaches to care in health service delivery has been identified as important to health care reform around the world. Many academic institutions have integrated interprofessional education (IPE) into curricula for pre-licensure students in healthcare disciplines, but few provide formal initiatives for interprofessional practice (IPP). It is recognized that experiential learning (EL) can play a significant role supporting IPP education initiatives; however, little is known of how EL is used within education for IPP in healthcare settings.

**Methods:**

We conducted a scoping review to map peer-reviewed literature describing IPP education initiatives involving EL for pre-licensure students in healthcare disciplines. A literature search was executed in MEDLINE, CINAHL, EMBASE, ERIC, PsycINFO, Scopus, and Social Services Abstracts. After deduplication, two independent reviewers screened titles and abstracts of 5664 records and then 252 full-text articles that yielded 100 articles for data extraction. Data was extracted using an Excel template, and results synthesized for presentation in narrative and tabular formats.

**Results:**

The 100 included articles represented 12 countries and IPP education initiatives were described in three main typologies of literature – primary research, program descriptions, and program evaluations. Forty-three articles used a theory, framework, or model for design of their initiatives with only eight specific to EL. A variety of teaching and learning strategies were employed, such as small interprofessional groups of students, team huddles, direct provision of care, and reflective activities, but few initiatives utilized a full EL cycle. A range of perspectives and outcomes were evaluated such as student learning outcomes, including competencies associated with IPP, impacts and perceptions of the IPP initiatives, and others such as client satisfaction.

**Conclusion:**

Few educational frameworks specific to EL have been used to inform EL teaching and learning strategies to consolidate IPE learning and prepare students for IPP in healthcare settings. Further development and evaluation of existing EL frameworks and models would be beneficial in supporting robust IPP educational initiatives for students in healthcare disciplines. Intentional, thoughtful, and comprehensive use of EL informed by theory can contribute important advances in IPP educational approaches and the preparation of a future health care workforce.

**Supplementary Information:**

The online version contains supplementary material available at 10.1186/s12909-024-05114-w.

## Introduction

Health care reform in Canada and around the world has emphasized the need for interprofessional, collaborative team-based approaches to delivery of health services that promote safer, integrated, coordinated, and holistic care to clients [1–3]. At the heart of this approach is interprofessional practice (IPP) that occurs when health providers from multiple different disciplines work together to provide high quality care to clients [3, 4]. Key to preparing a healthcare workforce for effective IPP is introduction of students in two or more health disciplines to both the theory and opportunities for collaboration and practice together to develop the competencies for IPP in their respective educational programs [5–7]. Of these elements, the practice component can be an effective educational strategy to provide students with meaningful experiential learning (EL) that consolidates competencies for interprofessional collaboration (IPC) through a combination of “hands-on” training, relational practice, opportunities to repeat experiences for learning, and reflection on one’s experiences [6–8]. The purpose of this article is to present findings of a scoping review that explored EL as an educational strategy for the preparation of students in healthcare disciplines for IPP in healthcare settings.

## Background

Effective IPP has been demonstrated to have a positive impact on client health outcomes through enhanced coordination of care and optimization of health service delivery [4, 9–11]. As such, a shift to IPP has become a focus in clinical practice, necessitating that it become a priority in health profession education. Many academic healthcare programs have incorporated elements of interprofessional education (IPE) and IPC into their curricula to build a foundation for IPP that introduces students to core competencies such as interprofessional communication, client-centered care, role clarification, team functioning, collaborative leadership, and conflict resolution [8, 12–14]. As defined by the World Health Organization, IPE “occurs when two or more professions learn about, from and with each other to enable effective collaboration and improve health outcomes” (pg. 13) [3]. IPE is considered an antecedent to IPC that “occurs when learners/practitioners, patients/clients/families and communities develop and maintain interprofessional working relationships that enable optimal health outcomes” (p. 6) [12].

Education strategies for the elements associated with IPE and IPC are often comprised of theoretical classroom instruction, client scenarios, role play, observation, simulation, case or interdisciplinary-based group activities, and related assignments [13–17]. As an example, until recently all first- and second-year students at the University of Manitoba in pre-licensure health sciences programs attended at least 12 synchronous events and participated in four asynchronous on-line activities over approximately 24 months to gain theoretical and initial exposure to IPE and IPC [18, 19]. In addition, all students were required to submit four written reflections on the role of IPC in their future practice over the course of the two years.

Despite the introduction of IPE and IPC experiences, few academic institutions offer formal learning initiatives within curricula for undergraduate students of different disciplines to work together in IPP practicums within their respective programs. This is due to barriers such as variations in class schedules, lack of formal collaborative leadership training in IPE champions (e.g., faculty and placement clinicians), challenges with finding clinical placements that support IPP, and inadequate administrative support [15, 20, 21]. While practical experience in IPP often can be gained by undergraduate students volunteering for extracurricular activities in student-led or student-run health initiatives in the community, many of these initiatives are outside of the formal curriculum without consistent professional mentorship, a guiding educational framework, or credit towards pre-licensure education [22]. Practical application and EL learning initiatives through IPP can help to consolidate knowledge gained through IPE and development of discipline-specific competencies [23].

**Fig. 1:**
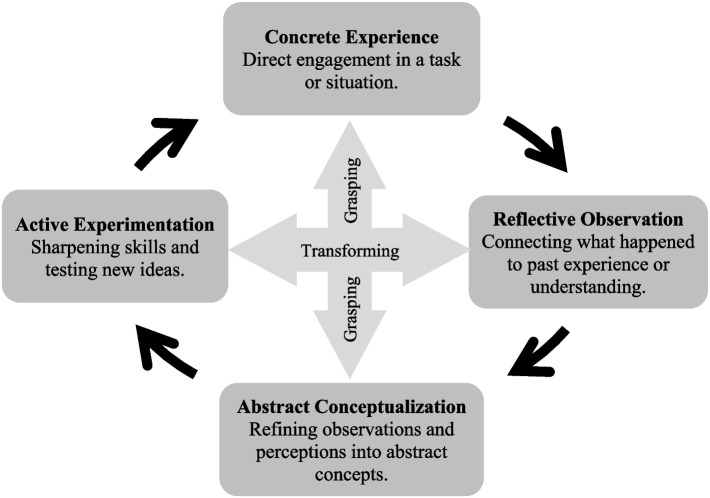
Kolb’s Experiential Learning Cycle (Kolb, 2015, [26] adapted from Long & Gummelt, 2020 [31])

*Experiential learning* is recognized as an effective teaching and learning strategy that helps learners to develop knowledge from real-life experiences [24, 25]. Although widely understood by many at a practical level as being “hands-on” learning by doing and knowledge from “real life” experience, EL was developed into a formal theory by Kolb informed by the works of John Dewey, Kurt Lewin, and Jean Piaget [24–28]. Kolb’s experiential learning theory (KELT) describes the role that experience plays in the learning process and is a dynamic, holistic and multi-dimensional approach to human development [26–29]. KELT recognizes that learning is best not viewed as an outcome but rather is continuous and grounded in experience [26–29]. Kolb defines *learning* as “the process whereby knowledge is created through the transformation of experience” (p. 49) [26] and results from the combination of grasping and transforming an experience through a cyclical sequence of stages that the learner progresses through in learning [26–29]. As such, foundational to KELT is the *experiential learning cycle* that consists of four modes and is recursive in nature: concrete experience, reflective observation, abstract conceptualization, and active experimentation (see Fig. 1) [26–29]. One model specific to IPE for students in health and social care that incorporates elements of KELT is the *Leicester Model of Interprofessional Education* that also has a 4-step education cycle designed for teaching interprofessional groups of students [30].

Our interest in understanding the use of EL as an educational approach to developing IPP competencies originated from foundational work to plan and implement student-infused community health initiatives as a formal component of curricula for students in healthcare disciplines [22]. Student-infused initiatives are requisite learning initiatives developed and offered by academic programs as part of curricula for students to meet entry-to-practice competencies through EL and/or service learning [22]. Since most pre-licensure students in healthcare programs at our university already participate in mandatory IPE and IPC course work and sessions foundational to IPP, our vision is to develop authentic learning experiences that facilitate meeting the requirements of their program and promote development of entry-to-practice competencies for both IPP and their discipline [14, 32–34]. To support this vision our aim is to develop evidence-informed IPP education initiatives based on KELT and best practices.

Use of EL or *experiential education*, a frequently used synonym for EL, and KELT to educate students in various healthcare disciplines has been reported in the literature and has been studied in relation to simulation exercises, acute clinical practice, service learning, and other contexts [17, 31, 35–37]. KELT has also specifically been used in relation to IPE and IPC as a theoretical framework to explore development and transformation of knowledge among healthcare students working together in interdisciplinary groups in case studies and simulated environments [38, 39]. To deliver an EL initiative that intentionally and thoughtfully brings multiple health disciplines together to learn with, from, and about each other in a clinical practice setting, it is important that faculty and clinical mentors have pedagogical strategies for students to experience and acquire competencies in IPP. A cursory exploration of the literature in CINAHL and PubMed did not identify existing systematic or scoping reviews specific to the integration of EL as a teaching strategy for IPP education initiatives. Thus, the goal of this scoping review was to identify and map what is known about EL as a teaching and learning strategy to prepare pre-licensure students of any level (e.g., baccalaureate, graduate, interns, etc.) for IPP in healthcare settings. The specific objectives were:

- To map and describe the context for EL in IPP education initiatives;
- To identify existing theories, frameworks, and models applied to education initiatives for IPP;
- To describe the teaching and learning strategies that facilitate IPP education in healthcare settings; and
- To determine what aspects of IPP EL have been evaluated and how evaluations were conducted.

## Methodology

A scoping review is considered an appropriate and useful means to: a) determine the extent and nature of the literature that is available on a specific topic; b) identify emerging evidence on the topic; and c) identify gaps in knowledge [40–42]. Given our goal to identify and map what is known about EL strategies that facilitate IPP education initiatives, a scoping review was a suitable methodological approach for our study. Informed by methods outlined by Arksey and O’Malley and the Joanna Briggs Institute, our approach to this scoping review consisted of four phases: a) literature search; b) title and abstract screening; c) full text screening; and d) data extraction and synthesis [40, 41, 43]. We also used the Preferred Reporting Item for Systematic Reviews and Meta-Analyses (PRISMA) to guide reporting of this scoping review [42, 44].

We defined IPP in pre-licensure education as being an opportunity where students of any level from at least two distinctly different healthcare disciplines worked together in collaboration to provide care and/or services as part of their learning. We recognized that the concept of IPP is often used synonymously and/or interchangeably with other terms, such as IPE, IPC, interprofessional collaborative practice (IPCP), and interprofessional learning (IPL). Therefore, similar to other systematic and scoping reviews, we incorporated these and other synonymous concepts when developing the literature search strategy [4, 8, 45]. For the purpose of this study, we defined *experiential learning* as any educational initiative where interprofessional groups of pre-licensure healthcare students work in a real-time practical (i.e., “hands-on”) environment to meet requirements and/or self-identified learning needs in the formation of interprofessional competencies.

### Literature search

**Fig. 2:**
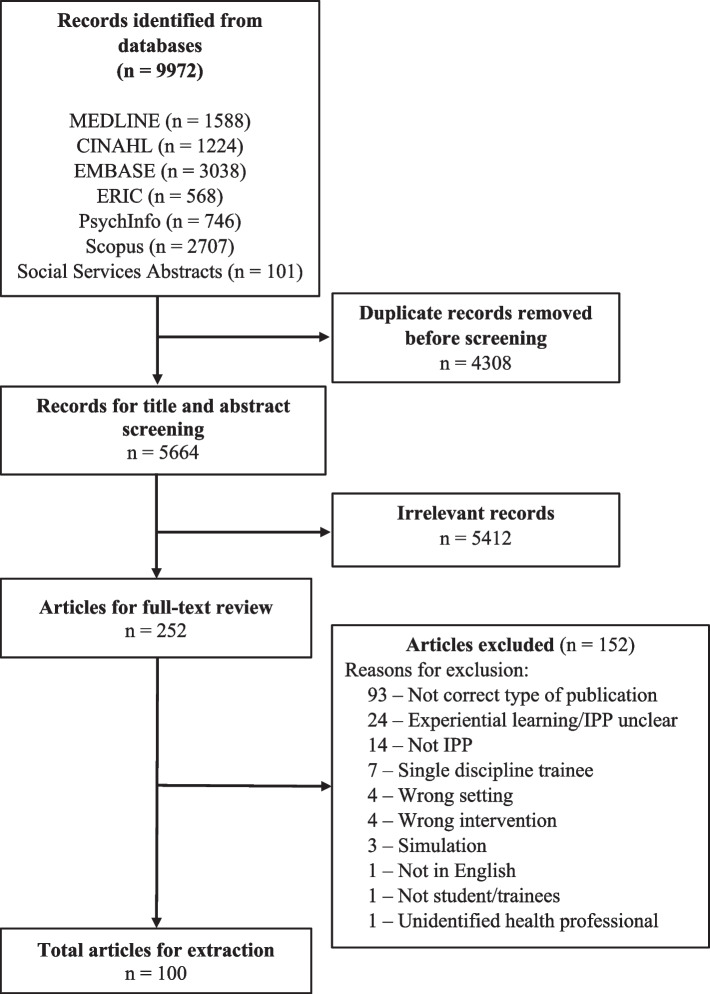
PRISMA diagram

The initial literature search strategy was conceptualized in consultation with a health sciences librarian experienced in systematic review searches and the strategy included combinations of index terms and key words related to IPP, health disciplines, education, and practice settings. A literature search strategy (see Supplementary file 1) was developed for MEDLINE (OVID) and peer-reviewed by another librarian using a procedure outlined by McGowan et al. [46]. This strategy was then then translated for CINAHL with Full-Text (EBSCOHost), EMBASE (OVID), ERIC (ProQuest), PsycINFO (OVID), Scopus, and Social Services Abstracts (ProQuest) and a full literature search was conducted in all seven databases to include peer-reviewed articles published from 2001 to July 11, 2023, and limited to records in English. The result was 9972 records that were first deduplicated in EndNote™ using the method developed by Bramer et al. [47] with additional duplicates being removed in Covidence™, a systematic review management system, to yield 5664 records for screening (see PRISMA diagram in Fig. 2).

### Title and abstract screening

Screening of titles and abstracts was independently completed by two reviewers in Covidence™ with conflicts being resolved by a third reviewer. The inclusion criteria for the scoping review were: a) a focus on experiential teaching or learning in a healthcare setting; b) IPP that included pre-licensure students from two or more distinctly different healthcare disciplines; c) peer-reviewed articles that represented primary research, program descriptions, program evaluations, and similar literature; and d) results that were available in English. A total of 252 records were identified for full text screening; however, 326 records were excluded due to missing abstract.

### Full text screening

Two reviewers independently reviewed full text articles using the same inclusion criteria previously noted for title and abstract screening. Records were excluded if: a) settings were simulation experiences rather than healthcare practice; b) citations reflected conference proceedings, poster presentations or reviews; and c) full text articles were unavailable. A third reviewer resolved conflicts.

### Extraction and synthesis of data

The principal leads of the research team (DAN, JLP, GH) developed a template for data extraction based on the goals and objectives of the scoping review, and to capture descriptive characteristics of the included articles (see Supplementary file 2). An Excel™ spreadsheet was created for data extraction and trialed using a selection of included articles. Template categories were refined through comparison and discussion of initial findings and the Excel™ spreadsheet was revised as data extraction progressed. Two reviewers worked in collaboration to extract data from each article with the first reviewer populating the Excel™ spreadsheet and the second reviewer verifying and adding any additional information. Where there was uncertainty about whether an article ought to be included or categorization of extracted data was unclear, at least two of the principal leads reviewed the conflicts and decided on inclusion or exclusion through consensus. Data were organized and presented in either tabular format or synthesized into a narrative summary as appropriate for the results being reported. Content and thematic analysis was used to create categories as headings in relation to the goal and objectives for this study.

## Results

Our scoping review resulted in 100 articles for data extraction and represented a range of examples where EL was used as an education strategy to prepare students from two or more disciplines for IPP. We provide a summary of final articles included from our scoping review in Supplementary file 3. We present the key findings from our review in three parts: 1) Part 1 provides an overall summary of descriptive elements of the included articles; 2) Part 2 provides the context of IPP education initiatives; and 3) Part 3 details the implementation and evaluation of IPP education initiatives. Given the total number of articles is 100, we report our findings in whole numbers that can be equated to percentage values.

### Part 1: overall summary of articles

In this section we provide a description and background from the included literature that details the: 1) descriptive elements of the articles; 2) variation of terminology related to IPP in education; and 3) aim or purpose of the articles.

#### Descriptive elements of articles

**Fig. 3:**
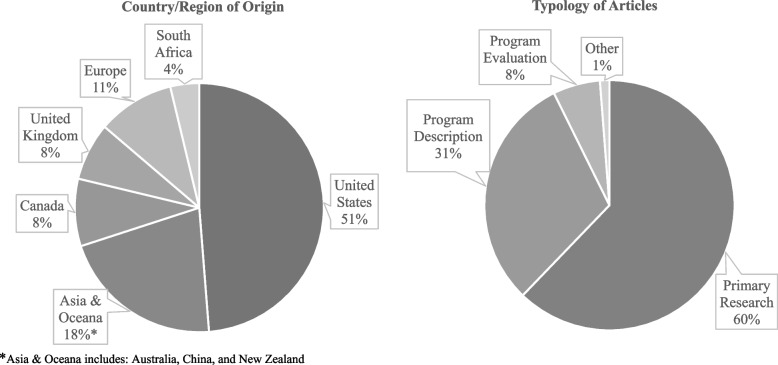
Articles by country/region of origin and typology of articles

The articles originated from 12 individual countries across four continents and described EL strategies for IPP education initiatives in three main typologies of literature – primary research, program description, and program evaluation (Fig. 3). The one article categorized as “other” was a practice guide focused on developing practice-based interprofessional learning [48]. Across those articles categorized as primary research and program evaluation, there was a range of methodological approaches used that included 25 quantitative, 25 mixed methods, 16 qualitative, and five case study designs (see Supplementary file # 2). Of note, there was one randomized control study [49] and five longitudinal studies identified [50–54].

#### Variation of terminology related to IPP in education

Our findings revealed that only two articles resulting from our search used *IPP* as the main concept in relation to IPP education initiatives in [55, 56], whereas the term *interprofessional collaborative practice* (IPCP) was used in five articles [50, 53, 57–59] and *interprofessional education and collaborative practice* was used in one article [60]. In 30 articles IPE was the sole concept used in association with IPP education initiatives. *Interprofessional learning* (IPL) was used in six articles [49, 61–65] and IPC in five articles [66–70] as synonymous terms for IPP. A range of other terms were used in conjunction with IPP including *interprofessional learners* [71], *interprofessional* (*collaborative*) *service-learning* [72–76], *interprofessional training* [77–79], *interprofessional* (*clinical*) *placement* [80–82], *interprofessional clinic experience* [83], *interdisciplinary student placements* [84]*, interdisciplinary student learning* [55], *interdisciplinary practice* [85], *interdisciplinary collaboration* [86], *interdisciplinary team* or teamwork [87], *integrated training* [88], *collaborative care training* [89, 90]*, collaborative practice* [88], *interprofessional workplace* [79], and *interprofessional global service-learning* [91].

#### Aim or purpose of the articles

The aim or purpose of the included articles was the explicitly stated or implied reason for why the paper was generated, which was often closely linked to the typology of the article (Fig. 3 and Supplementary file 2). Interpreting and reporting the typology of some articles posed a challenge as the aim or purpose was not explicitly stated [69, 70, 72, 91–99]. Where an explicit aim or purpose was not clear, a consensus how best to categorize the typology and describe the purpose was made by two or more members of our team from other information in the article. We identified three broad themes of purposes across the articles: a) describing and/or evaluating overall IPP initiative; b) evaluation of placements and experience on student outcomes; and c) experience, outcomes and/or impact of the IPP initiative on stakeholders (e.g., recipients of care, placement agencies, and collaborating partnerships). As some articles had more than one aim or purpose conveyed in the narrative or stated objectives these articles are represented in more than one theme.

- *Describing and/or evaluating the overall IPP initiative.* Fourty-four articles provided a description of planning, implementation and/or evaluation of elements of an IPP program [48–50, 57, 59, 60, 62, 65, 67, 69, 71, 72, 75, 78, 81, 87–91, 93, 96, 100–121], while the intent of one article was to describe the importance of IPP in relation to service delivery, care and meeting the needs of a population [122]. Program evaluation of the overall implementation or outcomes of an IPP initiative, such as curriculum design, was identified as a primary purpose in 13 articles [56, 59, 75, 95, 98, 114, 117, 123–128] and evaluation of impact of the IPP initiative on stakeholders (i.e., clients, health agency placements, etc.) was also identified [53, 59, 61, 62, 129]. Specifically introducing and/or evaluating the model, framework, or program design of the IPP initiative was a stated purpose [86, 118, 130, 131] and understanding processes of student learning through IPP curriculum design and experiences was also noted [132–134].
- *Evaluation of placements and experience on student outcomes*. Evaluation of the impact of clinical placements on students’ attitudes and/or perspectives towards IPE, IPC, IPL, IPP, and/or their placement experience was a purpose in 30 articles [49–52, 63, 65, 68, 72, 73, 76–78, 82, 83, 85, 98, 117, 118, 121, 122, 130, 135–143]. In 28 articles a purpose was to describe some specific aspect of the students’ perceptions on their experience, learning, or success from the IPP education initiative, such as competency development, cultural beliefs, interprofessional socialization, understanding of complex health issues, or other aspect of professional development [49–51, 54, 55, 58, 60, 66, 72, 74, 75, 79, 80, 97–99, 116, 118, 122, 123, 127, 130, 133, 142, 144–147]. Two articles evaluated student perspectives on their placement experience in relationship to their intentions to pursue a particular focus, such as working in primary care or rural settings [94, 139].
- *Impact of the IPP initiative on stakeholders*. The effect of IPP on client and service outcomes that included health status was a purpose in two articles [59, 62], while other articles aimed to evaluate experiences and perspectives of clients and their families on students engaged in IPP [50, 78, 84]. Six studies sought to assess the impact of the IPP initiative on clinical stakeholders and partnerships, such as effects of IPP on staff, collaboration or teamwork, practice efficiencies, and the health agency hosting placements [53, 59, 63, 64, 133, 148].

### Part 2: context of IPP Education Initiatives

This part provides the context of IPP education initiatives described in the literature and includes: 1) a general overview of the initiatives; 2) the intention of experiential learning in the initiatives; 3) nature of student participation in the initiatives; 4) populations served by the initiative; and 5) involvement of instructional personnel and stakeholders.

#### General overview of IPP Education Initiatives

In Table 1 we present a summary of the student disciplines involved in IPP education initiatives, the various settings in which the initiatives were situated, and the types of services students engaged in as part of their learning experience.

**Table 1:** Summary of student disciplines, settings and services provided represented in articles. (*n*=100)

**Student Discipline**	%^a^	**Settings**	%^a^	**Services**	%^a^
Medicine	71%	Primary healthcare (urban)	26%	Interviews/assessments	46%
Nursing	61%	Outpatient/Specialty clinic^d^	16%	Primary care interventions^h^	28%
Pharmacy	46%	Hospital (urban or rural)	16%	Prevention (education)	23%
Social work	29%	Community/agency site^e^	15%	Prevention (screening)	15%
Physical therapy	27%	Residential facilities^f^	10%	Involved in care planning	14%
Occupational therapy	25%	Public/community health	7%	Outreach/home visits	8%
Nutrition/Dietetics	20%	Academic/faculty sites	5%	Community-based projects	6%
Speech/language therapy	15%	Primary healthcare (rural)	5%	Made referrals for care	5%
Nurse practitioners	15%	Home visits	5%	Rehabilitation/exercise	4%
Dentistry	13%	Student-run clinic	5%	Navigation of services	1%
Physician assistant	12%	Schools/Education programs	4%		
Psychology (counselling)	9%	Mobile (rural)	2%	Not stated/unclear	20%
Total other pre-licensure health disciplines^b^	2%			Not applicable	1%
		Not stated/unclear^g^	10%		
Total other health-related disciplines^c^	20%	Not applicable	1%		
Disciplines unclear/unknown	3%				

^a^Percentage total exceeds 100% with disciplines, settings, and services being represented more than once in articles

^b^Total other health disciplines that appear in ≤5 articles such as midwifery, dental hygiene, pharmacy technician, respiratory therapy, etc.

^c^Total other health-related fields that appear in articles such as psychology, pre-medicine, public health, health administration, etc.

^d^Includes dental, pediatric, and other types of clinics

^e^Includes churches, shelters, non-profit agencies, and other non-specific community sites

^f^Includes long-term residents, extended care, and assisted living facilities

^g^Includes articles that provided a specific site or services, but also alluded to “other placement sites” or services without elaboration

^h^Primary care interventions include a range of activities (e.g., immunizations, medication administration, rehabilitation, counseling, dental, etc.)

#### Intention of experiential learning in IPP education initiatives

The intention of the EL IPP education initiatives was either explicitly stated or described by authors as the rationale for designing and implementing the initiative. Similar to our findings for purpose, several articles appeared to have more than one intention for the IPP initiative. In 22 articles, creation of learning initiatives for planned and/or authentic placements and experiences for interprofessional experiences was an intention of the IPP education initiative [51, 58, 62, 64, 67, 76, 78, 81, 85, 89, 95, 101, 103–105, 107, 117, 118, 120, 134, 139, 148]. Related to this, 20 of the initiatives were designed to build on IPE, IPL, and/or IPC theory or experiences through the initiative [48, 59, 70, 80, 87, 92, 106, 109, 111, 112, 114, 128, 131, 133, 135, 137, 138, 140, 141, 144]. Gaining familiarity with other disciplines and professions and learning from each other was cited as an intention in 16 of the IPP education initiatives [55, 56, 64, 65, 73, 74, 82, 88, 91, 98, 115, 121, 130, 134, 135, 141]. For 10 of the articles an intent was to provide IPP EL to pairings of specific student disciplines, such as: medicine and pharmacy [68, 121]; medicine and physician assistants [96]; medicine and psychology [90]; nursing and medicine [133]; nurse practitioner and dentistry [113]; nurse practitioner and medicine [146]; physical therapy and occupational therapy [75, 111]; and psychology interns and primary care physicians [89]. Creating IPP experiences and placements related to service-learning requirements or activities or as a required curriculum course was described in nine articles [58, 67, 74, 91, 97, 101, 110, 115, 121].

Demonstrating educational outcomes through IPP, such as acquisition of competencies related to IPC, discipline-specific competencies (e.g., assessments, interventions, clinical reasoning, etc.), and self-directed learning, was an intention in 26 of the articles [50, 51, 54–56, 60, 63, 75, 77, 78, 83, 84, 86, 91, 93, 95, 101, 102, 106, 108, 110, 116, 127, 130, 134, 135]. Gaining knowledge and being able to care for clients from diverse cultural, ethnic, racial, and/or other specific population groups (e.g., children, older adult, vulnerable, etc.) was identified as an intention for 10 of the IPP initiatives [49, 50, 75, 91, 99, 110, 117, 127, 145, 147]. In four articles an intention was to produce reflexive learners and critical thinking in their discipline roles [61, 73, 100, 111], while three aimed to develop interprofessional capacity in primary health care education [52, 108, 116] and five promoted faculty engagement in IPP or collaboration between education and health systems (e.g., academic partnerships) [99, 108, 123, 125, 148]. Developing, piloting, and/or evaluating a model of interprofessional delivery of service was an intention in seven articles [57, 62, 81, 101, 125, 136, 147].

Contributing to the community and creating awareness of the social determinants of health was an intention indicated for 11 of the IPP education initiatives, such as poverty and homelessness [62, 71, 76, 81, 85, 105, 119, 130, 143, 145, 147]. Improving the health care delivery system, addressing gaps in service delivery, addressing the needs of specific communities or populations, and improving care for clients were intentions described in 24 articles [59, 60, 71, 75, 76, 82, 84–86, 95, 99, 102, 104, 105, 112, 113, 117, 119, 126, 128, 133, 140, 145, 146], and in five of the articles the goal was production of a skilled workforce that included development of leadership in IPP teams [52, 82, 94, 123, 147]. Increasing the understanding, interest, and number of graduate practitioners in primary health care and rural settings were specific intentions of three IPP education initiatives [94, 108, 130]. Promoting an interprofessional ethos and client-/service user-centered care within a team were also identified as intentions of IPP education initiatives [57, 61, 133]. No explicit intention or purpose for the IPP education initiative was provided in six of the articles; authors in two of these articles refer to previous publications for details on the initiative [53, 66, 68, 90, 129, 132].

#### Nature of student participation

Nature of student participation reflected how students were involved in the IPP education initiative. For 41 IPP initiatives described in the literature the learning experience was a clinical placement where students of at least one discipline were assigned to participate as part of their educational program with other students from other disciplines participating through elective coursework or volunteerism [49, 53, 55, 56, 59, 62, 63, 68, 74, 77, 81–87, 93, 95, 96, 98, 103, 104, 106, 107, 109, 111–113, 115, 120, 123, 128, 131, 134, 135, 137, 141, 142, 146, 148]. Terms that were considered synonymous with clinical placement included *clinical rotation*, *training site*, *clerkship*, and *residency* [68, 77, 83, 86, 93, 112, 123]. In seven articles the initiative appeared to be required as part of course work or curriculum for at least one discipline [49, 61, 68, 98, 100, 112, 126] and in 10 instances it appeared to be an elective course or optional experience for credit for at least one discipline [50, 51, 54, 64, 71, 91, 101, 117, 119, 120]. Participation of students in IPP education initiatives was identified as voluntary in 15 articles [52, 60, 66, 69, 79, 97, 108, 114, 118, 122, 125, 127, 132, 138, 145] and three articles suggested either a voluntary nature or clinical practicum requiring an application process [76, 85, 130].

Many articles described a mix of participation models by students from different health disciplines. For example, in seven IPP education initiatives some students could pick either to be volunteers for the initiative or to have a clinical placement at the same initiative [68, 73, 105, 121, 126, 133, 136]. A second model of mixed participation was where some disciplines were mandated to the initiative for clinical practice while other disciplines were there as an elective/optional course or otherwise to receive credit [50, 58, 72, 101, 120, 146]. A third model of mixed participation was where it was voluntary for one discipline but either an elective or receipt of credit for other disciplines depending on program policies [51, 54, 91, 119]. In 19 articles the nature of student participation was either not stated or unclear.

#### Populations served by the initiative

In addition to a variety of settings where IPP education was situated (Table 1) the initiatives provided services to a range of population groups. By specific age group, services were provided to infants and children [49, 74, 103, 107, 113, 123, 126], adults [70, 81, 136], and older adults (≥60 years old) [50, 57, 66, 83–86, 105, 112, 116, 117, 146]. Services were provided to patients with specific conditions or disease processes that included chronic disease (e.g., COPD, diabetes, hypertension, HIV, etc.) [51, 53, 57, 59, 62, 65, 78, 80, 81, 92, 95, 111, 118, 121, 132, 141, 148], childhood obesity [73], dementia or cognitive decline [84], disabilities [49, 61, 83, 114], orthopedics [63, 137], and prenatal care [58]. Services were also provided to a mix of age and/or medical conditions, such as: complex and multiple health needs [50, 134]; primary school students and patients with dementia [135]; and varied patient groups or sites associated with an IPP initiative (e.g., diabetes, frail elderly, maternity, nursing homes, palliative care, etc.) [79, 106, 115, 134]. Populations were also defined by specific geographical location that included rural or isolated areas [80, 105, 108, 115, 130, 133, 139, 144, 148], other underserviced or deprived areas [100, 126, 143], and international location [76, 91]. Articles described populations as belonging to a minority, marginalized, vulnerable, uninsured, and/or otherwise socioeconomically disadvantaged group [51, 58, 60, 71, 72, 75, 76, 83, 89, 97, 101, 104, 115, 122, 127, 129, 139, 145, 147], having high utilization of healthcare resources [67], or being veterans [77, 93, 146]. No specific population was identified or clearly stated in 25 articles; however, many of these IPP education initiatives were situated in ambulatory care, primary care, or primary healthcare settings with a general population [77, 79, 89, 94, 98, 100, 102, 115, 125, 138] or residents of facilities, such as long term care [79, 99]. Population was not applicable in one article detailing a practice guide for IPE/IPP [48].

#### Involvement of instructional personnel and stakeholders

The majority of articles (*n* = 94) described some aspect of roles and participation of faculty, clinical instructors, site-based professional preceptors or mentors, and/or other stakeholders in the planning and operation of IPP education initiatives. There was variation in terminology used to describe instructional personnel participating from academic settings (e.g., academic educators, academic staff, clinical instructors, clinical supervisors, faculty members, lecturers, practice-educators, professors, university clinical facilitators, residents, teachers) [52, 61, 91, 94, 101, 103, 116, 123, 129, 131, 136, 140, 144, 148] and general use of terms to denote professional positions (e.g., facilitator, mentor, licensed clinicians, nurse educator, placement practitioner, preceptor, primary care preceptor, professional staff) [48, 56, 58, 61, 87, 89, 128, 135, 141], which made it challenging to distinguish whether instructional personnel were strictly from academic institutions or from the host setting. However, professional input to planning and oversight by licensed practitioners, whether from academic or host institutions, was a foundational element in supporting learning of pre-licensure students in IPP education initiatives.

Instructional personnel were involved in planning and coordination of IPP education initiatives that included collaborating on setting goals and objectives, developing activities, identifying community health needs, training practice educators, and setting policies for courses [48, 51, 52, 65, 74, 78, 92, 115]. Stakeholder involvement (e.g., administrators, health insurance planner, policy makers, staff, teachers) from the healthcare setting, service organizations, and other departments included participation in advisory boards, coordination of placements, leadership of clinical education, orientation, pursuit of research initiatives, and the public policy process [64, 74, 81, 95, 99, 102, 114, 129, 147, 148]. Instructional personnel provided or led seminars, educational support, reflective sessions, IP team meetings, reviewed assignments, and other non-clinical teaching activities [98, 101, 112, 118, 131, 140, 141]. Professional oversight of students in placements that was explicitly described was either solely by academic personnel [50, 52, 54, 57, 60, 62, 66, 76, 77, 83, 85, 88, 94, 103, 115, 120, 126, 127, 131, 136, 142, 146, 147] or with involvement of licensed personnel from the healthcare setting [51, 53, 56, 58, 59, 61, 63–65, 68–70, 72, 75, 82, 86, 87, 89–91, 97–99, 104, 106–108, 110–112, 114, 121, 122, 129, 130, 132–135, 137, 139, 141, 143, 144]. An interprofessional mix of disciplines for instructional personnel in program planning and experiences in clinical settings was clearly noted in much of the literature [51, 53, 54, 58, 59, 63, 66, 68, 74–76, 78, 81, 83, 88, 89, 101, 103, 110, 116, 125, 127, 133–137, 142, 146].

There were references to healthcare positions (e.g., clinical coordinator, community practitioner, health care assistant, health education coordinator, health worker, facilitator, IPE facilitator, laboratory staff, supervisor, teaching registrar, tutors) with direct involvement with students but whose professional credentials were unclear [49, 55, 56, 70, 73, 80, 87, 109, 111, 127, 130, 131, 134, 141, 143, 148]. The literature also highlighted involvement of other academic staff and stakeholder personnel, such as support staff (e.g., patient registration specialists, medical assistants, schedulers, etc.) [53, 59, 125, 126], project managers and administrators [102, 108], interpreters or translators [60, 72, 127], lay health promoters [60], teachers or teaching assistants [74, 119], and volunteers [60]. A few articles described IPC and IPP teaching with respect to experience and commitment of instructional personnel, particularly in placement sites, and recommendations were made related to preparation of instructional personnel to support IPP education initiatives [48, 56, 104, 108, 115, 134]. Anderson et al. provides a guide that “…offers a method for engaging clinical frontline practitioners in learning with, and from learners.” (p. 433) for practice-based IPE [48].

### Part 3: implementation and Evaluation of IPP Education Initiatives

This section details findings related to: 1) theories, frameworks, and models used in the design of IPP education; 2) teaching and learning strategies used in IPP education initiatives; and 3) evaluation of IPP EL.

#### Theories, frameworks, and models used to inform IPP education

Authors explicitly referred to using a theory, framework, or model to guide their approach in developing the IPP education initiative in 43 of the included articles (see Table 2). More than one theory, framework, and/or model was used to inform the educational approach in five articles [51, 57, 80, 117, 118]. Of these 43 articles, approximately 63% (*n*=27) were specific to education and behaviour. Eight articles used a theoretical framework and/or model for EL; two were based specifically on KELT [57, 146] and six were based on the Leicester Model that used Kolb’s work as a foundation [48, 61, 65, 100, 129, 131]. The application of a theory, model, or framework to support IPP education in six other articles was less clear as: a) the underpinnings were not explicitly stated; b) there was scant description of the application of stated theory or model directly to the educational approach (e.g., possibly linked to care delivery model); and/or c) it was difficult to distinguish whether the theory or framework was intended to guide the research approach rather than the educational initiative [60, 84, 97, 122, 130, 135]. There was no indication of a framework or theory to guide IPP education in 44 articles.

**Table 2:** Theory, models or frameworks for IPP Initiatives

**Category**	**Name**
Education and behavioural theories/models/ frameworks	Activity theory [132]
	Constructivist learning theory [127]
	Dewey’s education in action [117]
	Dutch framework for undergraduate medical education [112]
	EFECT model [50, 93]
	IPE or community-based IPE/interdisciplinary framework [85, 106, 136]
	Knowles’s principles of adult learning [57, 117]
	Kolb experiential learning theory [57, 146]
	Lave & Wenger’s sociocultural learning theory [80]
	Leicester model stages of learning [48, 61, 65, 100, 129, 131]
	Self-determination theory [79]
	Service-learning models [58, 75, 91, 101, 110, 143]
	Slavin’s six-stage model of group investigation [121]
	Wenger’s community of practice model [109, 134]
	Wenger’s concept of mutual engagement [80]
Frameworks or principles specific to IPC	Bronstein’s model of interdisciplinary collaboration [103]
	IPC core competencies/framework [51, 56, 74, 78, 80, 108, 118, 120, 123]
	IPL continuum [51]
Frameworks specific to care delivery and/or research	Adaptation of agency for healthcare research [92]
	Chronic care model/integrated care [57, 89]
	Clinical microsystems framework [125]
	Primary health care model [52]
Community-focused theory and frameworks	Community health empowerment model [147]
	Community interprofessional service learning model [73]
	Social capital theory [144]
	Social-ecological model and social determinants of health [118]

#### Teaching and learning strategies used in IPP education initiatives

A variety of approaches were used to facilitate the IPP education initiatives that were identified in this review. We broadly characterized these approaches as: a) *prerequisite and/or corequisite preparation; b)* s*pecific teaching and learning strategies; and c) time engaged in the IPP education initiative*.

- *Prerequisite and/or corequisite preparation.* Prerequisite preparation was characterized by a variety of activities that students were required to complete prior to engaging in the IPP education initiative. Prerequisite preparation was reported in 42 articles. This preparation was delivered through various modes such as: online courses and learning modules [53, 56, 59, 73, 74, 85, 118, 142]; in-person seminars, workshops, and meetings [49, 52, 55, 60, 64, 70, 72, 73, 75, 76, 82, 85, 88, 91, 99, 101, 106, 108, 110, 113, 123, 126–129, 136, 142, 145]; and individual readings and workbooks [64, 82, 90, 107, 118, 129]. The prerequisite training addressed a variety of topics including an introduction to interprofessional education or interprofessional collaborative practice [53, 56, 59, 60, 72, 76, 83, 99, 101, 103, 108, 113, 118, 123, 142], team building exercises [55, 76, 101, 102, 108, 118], and role expectations of students [52, 83, 99, 103, 118, 126]. Additionally, student orientation to the practice site was commonly a focus of the preparation [49, 52, 59, 64, 70, 76, 83, 85, 90, 108, 126, 145].

Corequisite preparation included some type of complementary learning activity that students were required to engage in at the same time as the IPP experience. Seven of the articles reported a corequisite component to the IPP education initiative [77, 87, 102, 106, 122, 133, 147]. For instance, entire courses or learning modules were intentionally developed and integrated throughout the initiative to complement the clinical experience [77, 87, 102, 133]. These were delivered in-person or online [102, 133], with a focus on such things as: interprofessional roles and responsibilities; effective communication and collaboration; case-based scenarios pertaining to the clinical site; behavioral change and leadership; and, service-learning more broadly [87, 122, 133, 147].

b. *Specific teaching and learning strategies.* Specific teaching and learning strategies included a wide range of methods and activities to facilitate student participation and support students through the learning process. A variety of specific teaching and learning strategies were presented within the IPP education initiatives included in this review. Having students intentionally work together in small interdisciplinary teams was explicitly reported in 62 of the articles. Teams varied in size, for example, from pairs [49, 56, 58, 59, 63, 65, 68, 108, 128, 137] to groups of three to seven students [70, 76, 78, 79, 85–87, 100, 102, 106, 115, 116, 121, 129, 140, 148, 149].

Throughout the experience, students engaged in various activities to facilitate IPP learning. Sixteen of the articles described implementing some type of intentional group discussion to start the day. For instance, team meetings [66, 102, 123], huddles [51, 53, 59, 81, 90, 94, 98, 102], and case conferences [146] were used to organize student groups, allocate client assignments, establish roles and responsibilities, and plan care. A variety of strategies in which students engaged with clients were also reported in 86 of the articles. For instance, students conducted assessments and developed care plans [51, 53, 55, 59, 67–70, 79, 84, 88, 99, 103–106, 112, 114–118, 123, 128, 131, 132, 136, 145, 148], delivered direct provision of care and services [50, 58, 63, 76, 78, 80, 81, 102, 107, 109, 120, 122, 126, 127, 134, 137, 141, 142, 146], provided home visits [49, 65–67, 86, 90, 98, 112, 117, 131, 140], developed and delivered health promotion or public health intervention or other education [52, 59, 72, 74, 85, 97, 101, 111, 119, 121, 147], engaged in outreach and other service activities [73, 75, 110], actively observed and shadowed healthcare professionals [55, 64, 90, 99, 138], engaged in team meetings or hub discussions [77, 78, 81, 82, 98, 115, 148], and delivered presentations to faculty and stakeholders [67, 77, 88, 93, 96, 105, 108, 111, 113, 115, 127, 130]. Debrief sessions or team meetings at the end of the day to discuss challenges, opportunities, and summarize the day’s experience were reported in 21 of the articles [51, 52, 57, 65–67, 70, 71, 76, 78, 81, 86, 92, 98, 102, 108, 110, 113, 118, 125, 128]. In addition, 23 of the articles explicitly reported the use of reflective activities to further help students process their responses to and learnings from the experience [57, 61, 63, 65, 71, 77, 78, 83, 87, 101, 106, 108, 110, 112, 115, 117, 118, 131, 134, 136, 139, 140, 145].

Unique strategies to promote team building throughout the educational experience were noted in five of the articles [85, 113, 126, 137, 144]. These included having shared accommodations, traveling together to the clinical site, and engaging in meals together as strategies for students and faculty to get to know one another, plan for the healthcare initiative, and engage in discussion before and after the day.

c. *Time engaged in the IPP education initiative.* The amount of time that students engaged in the IPP education initiative was reported in 76 of the articles. However, there was vast inconsistency in the way this was reported and a wide range of timeframes that were noted. For instance, some articles outlined the specific number of hours and/or days per week that students were engaged in the initiative [51, 54, 84, 103, 127] while others reported the overall length of the IPP initiative with little or no additional detail [49, 61, 62, 65, 75–77, 80, 96, 98, 99, 101, 106, 111, 113, 115–119, 129, 130, 134, 141, 148]. Overall, the experiences reflected a wide range of timeframes. For example, students may have engaged in a few hours or days [58, 64, 66, 78, 100, 104, 118, 121, 126, 127, 129, 131, 145, 146] up to multiple sessions over a number of weeks [49, 50, 65, 68, 75, 80, 92, 99, 102, 103, 117, 140]. Additionally, the length of engagement for different disciplines within a specific initiative often varied owing to variations in schedules between the departments [51, 53, 55, 59, 81–86, 93, 98, 105, 107, 109, 128, 133, 142–144].

#### Evaluation of IPP experiential learning

A range of perspectives and outcomes related to the experiential education initiatives were evaluated and presented in the literature. We broadly categorized these as: a) *evaluation of student learning outcomes*; b) e*valuation of IPP education initiatives*; and c) *other evaluated outcomes*. Various methods and approaches to evaluating and measuring a range of perspectives, learning outcomes, and the impacts of programs were described in the literature, particularly in the articles that reflected either primary research or program evaluation. As such, there were a number of articles that used methods and tools that crossed more than one of the aforementioned categories. For instance, Gruss and Hasnain used a mixed-methods approach to program evaluation that used the Interprofessional Education Collaborative Competency Self Efficacy Tool (IPECC-SET) to evaluate student learning outcomes related to IPC competency development and qualitative responses from students on the benefits of the IPP education initiative [117].

- *Evaluation of student learning outcomes.* Dimensions of student learning and professional growth were evaluated and reported in 35 articles. For instance, learning outcomes that were evaluated included competency development, acquisition of specific knowledge and skills, and performance of students who participated in IPP initiatives [50, 51, 57, 86, 98, 99, 108, 113, 114, 116, 117, 123, 126, 130]. Student perceptions and attitudes specific to IPE/IPC/IPP were also evaluated and included views of interdisciplinary practice, learning about interprofessionalism, and appreciation of own and other professional roles [49–52, 73, 77, 78, 85, 98, 118, 121, 125, 139, 145]. Similarly, perceptions of both students and stakeholders (e.g., preceptors or other involved clinicians) on IPE or IPP were measured [63, 108, 118]. Beliefs and attitudes towards IPP and collaboration were also evaluated [51, 54, 74, 83, 91, 108, 117, 118, 134], as were perceptions about interprofessional teamwork and the experience of workplace learning [51, 57, 79, 136]. Authors also reported changes of awareness and attitudes of students towards clients, the health care system, and care delivery while participating in IPP initiatives [99, 102, 105, 118].

There was a wide range of different tools and methods to evaluate student outcomes related to learning, competency development, perceptions of IPE/IPC/IPP, and other dimensions of their educational experience. The focus of evaluation and corresponding tools or methods used for evaluation are listed in Table 3.

**Table 3:** Evaluation tools and methods of student outcomes

**Focus of Evaluation**	**Tools or Methods**
Perceptions of/attitudes towards collaboration, teamwork or other professions	Assessment for Collaborative Environments (ACE-15) [50]
	Attitudes Toward Health Care Teams Scale (ATHCTS) [142]
	Focus groups [49, 51, 116]
	Interprofessional Collaborator Assessment Rubric (ICAR) [118]
	Interdisciplinary Education Perception Scale (IEPS) [59, 85, 98, 122, 142]
	Interprofessional Socialization and Values Scale (ISVS) [51, 54, 74, 141]
	Interviews [51, 66, 148]
	Questionnaire/survey (non-specific) [49, 70, 73, 91, 118, 138]
	Readiness for Interprofessional Learning Scale (RIPLS) [67, 78, 122, 127, 138]
	Scale of Attitudes Toward Physician-Pharmacist Collaboration (SATP2C) [68, 121]
	Students Perceptions of Interprofessional Clinical Education – Revised (SPICE-R2) [118]
	TeamSTEPPS® Teamwork Perceptions Questionnaire [57, 92]
Student learning outcomes (e.g., competency development, received knowledge, IP learning, etc.)	Case study [135]
	Caffrey Cultural Competence in Healthcare Scale (CCCHS) [127]
	Checklist of skills [102]
	Focus groups [61, 116, 143]
	Group debriefing sessions [76]
	Interprofessional Collaborative Competency Attainment Survey (ICCAS) [50, 51, 94, 99, 114, 123]
	Interprofessional Education Collaborative Competency Self Efficacy Tools (IPECC-SET) [117]
	Interviews [63, 82, 127, 137]
	Observation of students (e.g., preceptors, simulations, video sessions) [66, 95, 102, 113, 137]
	Questionnaire/survey [64, 73, 86, 100, 108, 126, 145, 146]
	Reflections/Reflective journals/notes [54, 76, 79, 91, 102, 114, 136, 148]
	Review of Interprofessional Competencies (RIPC) [51]
	Student Leadership Practices Inventory-Self (LPI) [147]
	Team Observed Structured Clinical Encounter (TOSCE) [54]
	Teams Skills Scale (TSS) [98]
	Tests/quizzes [113]
Change of student interest in area of practice/population focus	Attitudes Toward Community Health scale [147]
	Cultural Attitudes and Beliefs Scale (CABS) [118]
	Public Attitudes Towards Homelessness (PATH) scale [147]
	Questionnaire/survey (non-specific) [94, 105, 125, 139]
Preparedness to practice	Texas AHEC Survey (TexAS) [114]

b. *Evaluation of IPP education initiatives.* Evaluation of the IPP education initiative was reported in 32 of the articles and 15 of these were explicitly described as pilot studies [52, 62, 73, 74, 83, 87, 107, 108, 114, 125–127, 130, 133, 140]. The focus of evaluation was categorized into four main themes: a) perceptions of, and attitudes towards, the IPP experience and/or clinical experience; b) description and/or impact of learning in relation to the IPP education initiative; c) perceived value of the IPP education initiative and/or learning from it; and d) evaluation of design, components, instruction, and strategies of the IPP education initiative. The focus of evaluation and corresponding tools or methods used for evaluation or understanding of IPP education initiatives are listed in Table 4. The majority of articles identified as program evaluation used more than one scale and/or other method for evaluation [50, 75, 94, 117, 118, 123, 124, 128]; however, one study used only a survey of student’s experience of the practicum to evaluate the IPP education initiative [56]. One other study evaluated the perceptions of students towards IPE using the Interdisciplinary Education Perception Scale [52].

**Table 4:** Evaluation tools and methods of IPP education initiatives

**Focus of Evaluation**	**Tools or Methods**
Perceptions of/attitudes towards the IPP initiative/clinical experience	Focus groups [95, 112, 139]
	Interview questions [63, 82, 111, 133, 140]
	Questionnaire/survey [55, 64, 95, 117, 123, 131, 139, 140]
	Student Assessment of Learning Gains (SALG) [142]
	Students Attitudes Toward Community Service survey [97]
Description and/or impact of learning in relation to IPP initiative (e.g., acquisition of competencies, teamwork, communication, etc.)	Assessment for Collaborative Environments (ACE) [50]
	Entry-level Interprofessional Questionnaire (ELIQ) [59]
	Ethnographic observation of students [132]
	Focus groups [146]
	Interviews and photo-elicitation [144]
	Questionnaire/survey [130]
Perceived value of IPP initiative and/or learning from it (e.g., satisfaction of curriculum, sustainability, etc.)	Case study [135]
	Focus groups [61, 100]
	Questionnaire/survey [53, 64, 67, 86, 100, 130]
Evaluation of design, components, instruction, and strategies of IPP initiative.	Case study [134]
	Questionnaire/survey [87, 93, 100, 107]
	Self-Assessment of Clinical Reflection and Reasoning (SACRR) [122]

c. *Other evaluated outcomes.* Of note, there were other evaluative parameters reported that were not specific to EL but were related to either students’ roles in the services provided and/or tangential outcomes of the IPP education initiative. These included elements, such as client satisfaction in receipt of care, client perceptions of students, client health outcomes related to care through the IPP education initiative, stakeholder (e.g., preceptors, clinicians, agency representatives) perceptions of IPE/IPC/IPP, and other parameters [50, 53, 57–59, 61–64, 84, 93, 94, 101, 105, 107, 118, 128, 129, 131–133, 146, 148].

## Discussion

The goal of this scoping review was to identify and map what is known about EL as a teaching and learning strategy to prepare pre-licensure students for IPP in healthcare settings. In reviewing the literature, we identified 43 articles that utilized theories, frameworks, and models to plan and support implementation of IPP education initiatives; 27 of these were specific to education and behaviour. Eight articles explicitly referenced an EL theory or model (i.e., KELT, Leicester Model); however, several articles described initiatives that aligned with various aspects of EL theory. In addition, we identified and mapped specific teaching and learning strategies used in IPP education, as well as the way student learning outcomes and the overall initiatives were evaluated. For this discussion, we focus on a few articles identified in Table 2 that incorporated elements of Kolb’s work or described attributes that illustrated potential application of Kolb’s experiential learning cycle since KELT is widely recognized and has been adapted in various education contexts [24, 27, 31]. We also describe how other frameworks, theories, and models may be of benefit in augmenting EL learning experiences for IPP education. Finally, we offer further insight and better understanding of how EL can be more explicitly adapted and formally applied in the design of IPP education initiatives.

### Initiatives explicitly based on experiential learning theory or models

As noted in Table 2, KELT and the corresponding model were only cited in two articles [57, 146]. This was surprising to us as KELT was developed in 1984 and has been widely utilized for experiential education in many contexts outside of healthcare (e.g., business, higher education, service-learning, etc.) [28, 150], but has also been applied in IPE and other contexts of healthcare education [31, 39]. Five other articles cited use of the Leicester Model of IPE that adapted components of Kolb’s experiential learning cycle [61, 65, 100, 129, 131] and, similar to Kolb’s work, also incorporated elements of Piaget’s constructivist learning theory [151, 152]. Dewey’s work that is foundational to KELT was cited in one article [117], while one other used the EFECT model [93], a framework that is, in part, also adapted from Kolb’s experiential learning cycle [153]. However, not all these education initiatives appear to have used the full Kolb’s experiential learning cycle or repeated the cycle with a recursive element as intended for the KELT model to continue the EL process over time [26, 29].

For instance, Dolce et al. detail the concrete experience of a 4-hour clinical rotation of nurse practitioner and dentistry students conducting patient/family encounters together, then completing electronic documentation of the session, real-time feedback to each other, and a 30-minute debriefing session [57]. The joint patient/family encounter would be considered the concrete experience in KELT, and the feedback and debriefing in this example may be considered part of reflective observation. However, it is not clear how the modes of abstract conceptualization and active experimentation might have been incorporated or whether an opportunity to repeat the cycle was provided.

In another article, Mecca et al. provide a detailed conceptualization as to how they used KELT in an educational intervention designed for internal medicine residents and nurse practitioners that included strategies of learning activities, pre-clinic conference, and joint appointments with clients over two or three clinics [146]. The joint appointment in this example would likely be considered the concrete experience. The learning activities and pre-clinic conference may be considered active experimentation or potentially a dimension of abstract conceptualization; however, their relationship to the KELT modes is unclear. The design of this intervention did permit students to repeat the EL opportunity more than once and might lend itself well to providing a wholesome EL process with refinement to explicitly define and integrate the KELT modes.

Application of the Leicester Model of Interprofessional Education with a detailed account of student engagement throughout the learning cycle was well-described in two articles co-authored by developers of the model [61, 129]. Each of these articles described advanced preparation of students for the IPP experience that included a module handbook that emphasized IPC learning and also the integration of teaching and learning strategies to support the steps in the Leicester Model including work in small interdisciplinary teams, reflective exercises, and support by professional facilitators [61, 129]. However, it was unclear whether there was a singular encounter with clients in both instances and whether the learning cycle was repeated more than once. Toth-Pal et al. describe a similar application of the Leicester Model comprised of a singular one hour home visit with a client by a small interdisciplinary group of students supported by a clinical supervisor that was followed by discussion of the case, development of a care plan, and sharing of reflections [131]. The home visit clearly aligns with the concrete experience of Kolb’s experiential learning cycle and the sharing of reflections would seem to fit with reflective observation; however, the relationship of the other activities to steps of the Leicester Model is not clear and the total duration of the learning activity was only one hour. Of particular note, this IPP learning experience was also preceded with preparation through a client case description and students deciding on their responsibilities in advance of their home visit that can arguably be appreciated as part of the students’ learning but is not represented in KELT [131].

### Exemplars with foundations for experiential learning

Mann et al. presented “Seamless Care”, a robust experiential education initiative based on the *Interprofessional Education for Collaborative-Patient-centred Practice* (IECPCP) model developed by D’Amour and Oandasan [106, 154]. While not explicitly an EL theory or model, the IECPCP model has theoretical underpinnings that are similar to KELT (e.g., constructivist learning theory) and the “Seamless Care” initiative incorporated various teaching and learning strategies to support collaborative practice, such as interprofessional student practice groups, problem-based learning (i.e., interaction with clients), opportunities for reflection and integration of learning, and cooperative learning [106, 154]. Although not specifically based on Kolb’s work, the instructional strategies of the “Seamless Care” model appear to align well with the first two modes of Kolb’s experiential learning cycle. Specifically, the interaction with clients within mixed discipline student teams is a concrete experience from which students then enter the reflective observation mode through opportunities for reflection and integration of learning [106]. The cooperative learning in this initiative included problem-based learning that may have been potential opportunity for students to engage in abstract conceptualization and active experimentation; however, there was not enough information to make this determination [26, 28, 106].

Two other articles that also used an IPE or interdisciplinary practice orientation for their IPP education initiative applied competency frameworks for IPP, one employing the *Core Competencies for Interprofessional Practice* [136] and the other an original conceptual framework based on recommended concepts from a national advisory committee [85]. In both cases, students of different disciplines were provided concrete learning experiences interacting with clients under guidance of faculty or clinical supervisors and engaged in planned opportunities for reflection on the experience occurred; one group wrote a 500-word reflection related to the competencies [136] and the other used travel time together to discuss care delivery [85]. Although not designed with KELT in mind, both frameworks outlined here partially align with the first two modes of the EL cycle (i.e., concrete experience and reflective observation) and have the potential to be more fully developed as an EL IPP education initiative with the addition of teaching and learning strategies to round out the cycle [26, 28, 85, 136].

### Other theories that support experiential learning

Of the other theories, frameworks, and models used to inform the approach to IPP education initiatives, application of Wenger’s community of practice model and service-learning models supported EL [109, 134]. The main principle to Wenger’s model is focused on the development of professional identity through relationships and activities within a practice environment that, in the case of two particular articles, occurred in clinical areas where students worked together in interprofessional groups over a period of time (from one or two days to several weeks or an academic year) with the support of a mix of licensed healthcare providers [109, 134]. Teaching and learning strategies employed in these initiatives included in-service sessions covering IPE/IPC principles and knowledge related to client care, IPE tutorials, working together to provide care to clients, and participation in reflexive seminars [109, 134]. These elements and the described sequencing over the clinical placement time bear similarities to Kolb’s experiential learning cycle.

Where service-based learning or service-learning models were explicitly identified, a specific model or description of service learning was provided in five articles [75, 91, 101, 110, 143]. Flinn et al. provided a detailed literature review on service-learning and described it as a type of EL that is differentiated by having goals for both a commitment to service provision that meets community needs and student learning that incorporates reflection on service experience to learning objectives [75]. A similar approach to using service-learning as a teaching and learning strategy was described in four other articles [58, 91, 101, 110], and one article highlighted a service-learning design model based on the work of Gerda Bender that detailed a step-wise approach to curriculum design [143]. Each of the articles offered a practical, real-time educational initiative for students of different disciplines to learn together as a concrete experience but there were varied accounts of how reflection on, or cyclical application of, learning occurred throughout the IPP education initiative [58, 75, 91, 101, 110, 143]. Service learning has been associated with EL in our review and external literature, presented as a means to create a transformative experiential educational initiative with contributions of students usually to community (e.g., civic engagement, provision of service, etc.) and as a learning framework [17, 31, 35, 75, 91, 101, 110, 143].

### Recommendations and future directions

Our scoping review revealed that KELT and other frameworks or theoretical underpinnings have been utilized in various degrees to facilitate EL in IPP. Although there were gaps in describing application of all modes in the learning cycle and/or lack of clarity whether the learning cycle had been repeated to consolidate learning over time, these articles provide a starting point in understanding application of EL theory to development of authentic “hands-on” IPP education initiatives. By drawing on our findings of specific teaching and learning strategies and other exemplars in the literature external to our scoping review, it is possible to more fully develop theories and models, such as KELT, for intentional design of IPP education initiatives. For instance, Fewster-Thuente and Batteson describe all modes of KELT in the design and evaluation of a simulated IPE activity using a case study to explore attitudes and behaviours of interdisciplinary groups of pre-licensure students in healthcare programs [39]. Two other examples in the literature more fully explicate application and evaluation of KELT for students in other interprofessional healthcare contexts included settings specific to older adults in long term care and raising the public’s awareness of rights and responsibility in the healthcare system [31, 38].

With respect to theory and/or model testing for IPP education, this was illustrated in application of the Leicester Model with emphasis on its utility in facilitating student learning outcomes, such as preparation for professional practice and competency development [61, 100, 129]. However, few studies of IPP education initiatives have focused on all aspects of KELT and modes of its learning cycle; as highlighted in our preceding discussion there are rich sources in the literature to support further development and evaluation of KELT in IPP education. One specific area to evaluate is repetition of KELT learning cycles over time to explore the impact of duration in reinforcing and consolidating IPP learning outcomes.

Noting the dearth in longitudinal research and robust study designs for EL in IPP education initiatives, there is much opportunity to further knowledge development in this area. As evidenced in our review, there have been many pilot studies completed for IPP education initiatives and evaluation of a wide variety of outcomes, both learning and program, that can serve as a foundation for design, implementation, and evaluation of feasible and effective IPP education initiatives for pre-licensure students.

### Limitations

There are some limitations with our scoping review, foremost the challenge with developing a literature search given the range of concepts associated with IPP and variation in contexts where EL might occur with two or more pre-licensure health disciplines. As described in our findings there were approximately 19 different terms used synonymously and/or interchangeably with IPP; this inconsistency combined with the complexities of indexing in databases (e.g., limited controlled vocabulary, automated indexing, and indexing delay) meant it is likely that some articles might have been missed. For example, two articles we used in our background specific to KELT were not identified in our literature search even though they both described IPP for pre-licensure students [31, 39]. Related to this, how EL or similar concepts were described or used in education of pre-licensure healthcare students was complex with the same issues in lack of common terms, definitions, and indexing. From our experience with this review, it is important for researchers to recognize these limitations and to work closely with an experienced librarian to determine the breadth of subject headings and keywords for both design of literature search strategies and accurate indexing for published literature.

With respect to data extraction and analyzing our findings, another challenge we had was that many articles were not explicit in their description of the IPP education initiative, did not fully describe teaching and learning strategies, and were missing some of the contextual data we were seeking (e.g., education level of the learners, breakdown of disciplines, length of engagement, etc.). This was not critical given the nature of a scoping review is to map what is provided but did limit comparison of initiatives across studies. Finally, as evidenced by the concentration of articles from North America, our limitation to English articles may have excluded representation from Europe, South America, and other areas.

## Conclusion

To strengthen health care reform that is increasingly focused on interprofessional, collaborative team-based approaches to delivery of health services, it is imperative that robust and comprehensive educational strategies be developed to prepare pre-licensure students in competencies for IPP. Experiential learning can play an important role in this educational endeavour. Our scoping review has identified and mapped theoretical underpinnings, teaching and learning strategies, and various methods to measure both student learning and program outcomes from the literature that can serve as a foundation for the design, implementation, and evaluation of robust IPP education initiatives that include EL. While KELT and the Leicester Model have been identified as very specific theoretical frameworks with models to guide planning of IPP education initiatives there is much opportunity to build on these or envision other approaches to enhancing EL for students in healthcare disciplines. For example, combining either KELT or the Leicester Model with select teaching and learning strategies identified from our review can be adapted to a range of contexts and settings to provide pre-licensure students with a meaningful EL experience. Intentional, thoughtful, and comprehensive use of EL informed by theory can contribute important advances in IPP educational approaches and the preparation of a future health care workforce that is competent in high-quality, interprofessional, team-based care.

### Supplementary Information

**Additional file 1.** Literature Strategies and Results for Scoping Review.

**Additional file 2.** Data Points for Extraction.

**Additional file 3.** Articles Included for Extraction from Literature Search of Interprofessional Practice (IPP) Education Initiatives.

## Data Availability

All data generated or analyzed during this study are included in this published article and its supplementary information files.

## Abbreviations

EL: Experiential learning
IPC: Interprofessional collaboration
IPCP: Interprofessional collaborative practice
IPE: Interprofessional education
IPL: Interprofessional learning
IPP: Interprofessional practice
KELT: Kolb’s experiential learning theory
PRISMA: Preferred reporting item for systematic reviews and meta-analyses
