# Supplementary material for: Exploring experiential learning within interprofessional practice education initiatives for pre-licensure healthcare students: a scoping review
Source: BMC Med Educ. 2024 Feb 13;24:139. doi: 10.1186/s12909-024-05114-w (PMC10863283; doi:10.1186/s12909-024-05114-w)
Supplement: Supplementary file 1 — Additional file 1. Literature Strategies and Results for Scoping Review. [file 12909_2024_5114_MOESM1_ESM.pdf]

Supplementary File #1

Literature Strategies and Results for Scoping Review

Table 1. Medline

Database: MEDLINE(R) ALL <1946 to July 10, 2023><br>Platform: OVID<br>Date Searched: Jul 11, 2023		
#	Searches	Results
1	(clerkship* or preceptorship* or traineeship* or placement* or exposure* or practicum* or "off service" or fieldwork or "field-work" or "field training" or "field study" or "field studies" or "field education" or "field experience" or "workplace learn*" or "workplace training" or "practice learn*" or "practice based learn*" or "practical learn*" or "experiential learn*" or "experiential education" or "work based learn*" or "work integrated learn*" or "service learning" or "deliberate practice" or rotation or rotations or "clinical practice" or "clinical environment*" or "clinical setting*" or "clinical education" or "co-op" or "cooperative education" or "collaborative learning").mp.	1777873
2	Interdisciplinary Placement/	305
3	Clinical Clerkship/	5743
4	Preceptorship/	5616
5	or/1-4 [Practicum Concept]	1777873
6	(interprofession* or "inter-profession*" or interdisciplin* or "inter-disciplin*" or interinstitut* or "inter-institut*" or interagenc* or "inter-agenc*" or intersector* or "inter-sector*" or interdepartment* or "inter-department*" or interoccupation* or "inter-occupation*" or interorgani?ation* or "inter-organi?ation*" or multiprofession* or "multi-profession*" or multidisciplin* or "multi-disciplin*" or multiinstitut* or "multi-institut*" or multiagenc* or "multi-agenc*" or multisector* or "multi-sector*" or multidepartment* or "multi-department*" or multioccupation* or "multi-occupation*" or multiorgani?ation* or "multi-organi?ation*" or transprofession* or "trans-profession*" or transdisciplin* or "trans-disciplin*" or transagenc* or "trans-agenc*" or transsector* or "trans-sector*" or transdepartment* or "trans-department*" or transoccupation* or "trans-occupation*" or transorgani?ation* or "transorgani?ation*" or "cross-training" or crossprofession* or "cross-profession*" or crossdisciplin* or "cross-disciplin*" or crossinstitut* or "cross-institut*" or crossagenc* or "cross-agenc*" or crosssector* or "cross-sector*" or crossdepartment* or "cross-department*" or crossoccupation* or "cross-occupation*" or crossorgani?ation* or "cross-organi?ation*" or team* or collaborative or IPP or IPE or ILP or IPECP).mp.	552683
7	interprofession*.jw.	2316

8	Interprofessional Relations/	53342
9	Interprofessional Education/	459
10	exp Patient Care Team/	72815
11	or/6-10 [Interprofessional Concept]	552818
12	((primary or ambulatory or communit* or general or family) adj2 (care or healthcare or "health care" or "health program*" or "health centre*" or "health center*" or "health service*" or practice* or clinic* or practition* or medicine or doctor* or physician* or clinician*)).mp.	548437
13	("private pract*" or GP or PCP or CBHW).mp.	82406
14	(community or "general practice*" or "general practitioner*" or "family practic*" or "family medicine" or "primary care").jw.	121047
15	Community Health Services/	33119
16	Community Medicine/	2078
17	Primary Health Care/	91529
18	General practice/ or Family practice/	78544
19	Private Practice/	8502
20	exp Ambulatory Care Facilities/	59177
31	limit 30 to (yr="2001 -Current" and english) [FINAL set; with limits applied]	1588

Table 2. CINAHL

Database: CINAHL with Full-text<br>Platform: EbscoHost<br>Date Searched: Jul 11, 2023		
#	Searches	Results
S1	(clerkship* or preceptorship* or traineeship* or placement* or exposure* or practicum* or "off service" or fieldwork or "field-work" or "field training" or "field study" or "field studies" or "field education" or "field experience" or "workplace learn*" or "workplace training" or "practice learn*" or "practice based learn*" or "practical learn*" or "experiential learn*" or "experiential education" or "work based learn*" or "work integrated learn*" or "service learning" or "deliberate practice" or rotation or rotations or "clinical practice" or "clinical environment*" or "clinical setting*" or "clinical education" or "co-op" or "cooperative education" or "collaborative learning")	413,434
S2	(MH "Student Placement")	5,573
S3	(MH "Education, Clinical") OR (MH "Fieldwork")	15,370
S4	(MH "Preceptorship")	4,909
S5	S1 OR S2 OR S3 OR S4	416,008
S6	(interprofession* or "inter-profession*" or interdisciplin* or "inter-disciplin*" or interinstitut* or "inter-institut*" or interagenc* or "inter-	285,457

	agenc*" or intersector* or "inter-sector*" or interdepartment* or "inter-department*" or interoccupation* or "inter-occupation*" or interorgani?ation* or "inter-organi?ation*" or multiprofession* or "multi-profession*" or multidisciplin* or "multi-disciplin*" or multiinstitut* or "multi-institut*" or multiagenc* or "multi-agenc*" or multisector* or "multi-sector*" or multidepartment* or "multi-department*" or multioccupation* or "multi-occupation*" or multiorgani?ation* or "multi-organi?ation*" or transprofession* or "trans-profession*" or transdisciplin* or "trans-disciplin*" or transagenc* or "trans-agenc*" or transsector* or "trans-sector*" or transdepartment* or "trans-department*" or transoccupation* or "trans-occupation*" or transorgani?ation* or "transorgani?ation*" or "cross-training" or crossprofession* or "cross-profession*" or crossdisciplin* or "cross-disciplin*" or crossinstitut* or "cross-institut*" or crossagenc* or "cross-agenc*" or crosssector* or "cross-sector*" or crossdepartment* or "cross-department*" or crossoccupation* or "cross-occupation*" or crossorgani?ation* or "cross-organi?ation*" or team* or collaborative or IPP or IPE or ILP or IPECP)	
S7	(MH "Interprofessional Relations")	28,642
S8	(MH "Education, Interdisciplinary")	7,049
S9	(MH "Multidisciplinary Care Team")	50,261
S10	S6 OR S7 OR S8 OR S9	285,457
S11	((primary or ambulatory or communit* or general or family) N2 (care or healthcare or "health care" or "health program*" or "health centre*" or "health center*" or "health service*" or practice* or clinic* or practition* or medicine or doctor* or physician* or clinician*))	321,655
S12	("private pract*" or GP or PCP or CBHW)	57,766
S13	(MH "Community Health Services")	24,548
S14	(MH "Community Medicine")	349
S15	(MH "Primary Health Care")	72,202
S16	(MH "Family Practice")	26,195
S17	(MH "Private Practice")	9,055
S18	(MH "Ambulatory Care Facilities")	9,173
S19	(MH "Ambulatory Care")	13,368
S20	(MH "Physicians, Family")	22,866
S21	(MH "Community Health Nursing")	26,791
S22	S11 OR S12 OR S13 OR S14 OR S15 OR S16 OR S17 OR S18 OR S19 OR S20 OR S21	373,036
S23	(student* or learner* or resident or residents or intern or interns or PGY or graduate* or postgraduate* or "post-grad*" or undergraduate* or "under-grad*" or trainee*)	396,972
S24	(MH "Students, Health Occupations+")	91,435

S25	(MH "Education, Dental") OR (MH "Education, Nursing, Graduate") OR (MH "Education, Medical") OR (MH "Education, Nursing, Baccalaureate+") OR (MH "Education, Nursing") OR (MH "Education, Nursing, Associate") OR (MH "Education, Nursing, Diploma Programs") OR (MH "Education, Pharmacy")	102,625
S26	S23 OR S24 OR S25	444,310
S27	S5 AND S10 AND S22 AND S26	1,372
S28	S5 AND S10 AND S22 AND S26 [limit to english]	1,331
S29	S5 AND S10 AND S22 AND S26 [limit 2001 - present]	1,224

Table 3. EMBASE

Database: Embase <1974 to 2023 July 10><br>Platform: OVID<br>Date Searched: Jul 11, 2023		
#	Searches	Results
1	(clerkship* or preceptorship* or traineeship* or placement* or exposure* or practicum* or "off service" or fieldwork or "field-work" or "field training" or "field study" or "field studies" or "field education" or "field experience" or "workplace learn*" or "workplace training" or "practice learn*" or "practice based learn*" or "practical learn*" or "experiential learn*" or "experiential education" or "work based learn*" or "work integrated learn*" or "service learning" or "deliberate practice" or rotation or rotations or "clinical practice" or "clinical environment*" or "clinical setting*" or "clinical education" or "co-op" or "cooperative education" or "collaborative learning").mp.	2661821
2	collaborative learning/	1895
3	clinical education/	15430
4	or/1-3 [Practicum Concept]	2661821
5	(interprofession* or "inter-profession*" or interdisciplin* or "inter-disciplin*" or interinstitut* or "inter-institut*" or interagenc* or "inter-agenc*" or intersector* or "inter-sector*" or interdepartment* or "inter-department*" or interoccupation* or "inter-occupation*" or interorgani?ation* or "inter-organi?ation*" or multiprofession* or "multi-profession*" or multidisciplin* or "multi-disciplin*" or multiinstitut* or "multi-institut*" or multiagenc* or "multi-agenc*" or multisector* or "multi-sector*" or multidepartment* or "multi-department*" or multioccupation* or "multi-occupation*" or multiorgani?ation* or "multi-organi?ation*" or transprofession* or "trans-profession*" or transdisciplin* or "trans-disciplin*" or transagenc* or "trans-agenc*" or transsector* or "trans-sector*" or transdepartment* or "trans-department*" or transoccupation* or "trans-occupation*" or transorgani?ation* or "transorgani?ation*" or "cross-training" or	720010

	crossprofession* or "cross-profession*" or crossdisciplin* or "cross-disciplin*" or crossinstitut* or "cross-institut*" or crossagenc* or "cross-agenc*" or crosssector* or "cross-sector*" or crossdepartment* or "cross-department*" or crossoccupation* or "cross-occupation*" or crossorgani?ation* or "cross-organi?ation*" or team* or collaborative or IPP or IPE or ILP or IPECP).mp.	
6	interprofession*.jx.	2471
7	interprofessional education/	926
8	multidisciplinary team/ or collaborative care team/	28724
9	or/5-8 [Interprofessional Concept]	720198
10	((primary or ambulatory or communit* or general or family) adj2 (care or healthcare or "health care" or "health program*" or "health centre*" or "health center*" or "health service*" or practice* or clinic* or practition* or medicine or doctor* or physician* or clinician*)).mp.	710177
11	("private pract*" or GP or PCP or CBHW).mp.	146483
12	(community or "general practice*" or "general practitioner*" or "family practic*" or "family medicine" or "primary care").jx.	120331
13	community care/	60271
14	community medicine/	3017
15	primary health care/ or primary medical care/	209522
16	general practice/	85187
17	private practice/	18134
18	ambulatory care/ or ambulatory care nursing/	41745
19	general practitioner/	118696
20	community health nursing/	24217
21	or/10-20 [Primary Care Concept]	905886
22	(student* or learner* or resident or residents or intern or interns or PGY or graduate* or postgraduate* or "post-grad*" or undergraduate* or "under-grad*" or trainee*).mp.	973077
23	exp health student/	141107
24	medical education/ or dental education/ or physician assistant education/ or residency education/ or nursing education/ or nurse midwifery education/ or nurse training/ or pharmacy education/	383969
25	or/22-24 [Student Concept]	1199033
26	and/4,9,21,25 [FINAL SET; no limits]	3334
27	limit 26 to (english language and yr="2001 -Current") [FINAL Set; with limits applied]	3038

Table 4. ERIC

Database: ERIC<br>Platform: ProQuest<br>Date Searched: Jul 11, 2023		
#	Searches	Results
**S1**	(clerkship* or preceptorship* or traineeship* or placement* or exposure* or practicum* or "off service" or fieldwork or "field-work" or "field training" or "field study" or "field studies" or "field education" or "field experience" or ("workplace learning") or "workplace training" or ("practice learning") or "practice based learn*" or ("practical learning") or ("experiential learning") or "experiential education" or "work based learn*" or "work integrated learn*" or "service learning" or "deliberate practice" or rotation or rotations or "clinical practice" or ("clinical environment" OR "clinical environments") or ("clinical setting" OR "clinical settings") or "clinical education" or "co-op" or "cooperative education" or "collaborative learning")	109504
**S2**	MAINSUBJECT.EXACT("Adult Vocational Education") OR MAINSUBJECT.EXACT("Cooperative Education")	4760
**S3**	MAINSUBJECT.EXACT("Clinical Experience") OR MAINSUBJECT.EXACT("Clinical Teaching (Health Professions)") OR MAINSUBJECT.EXACT("Internship Programs") OR MAINSUBJECT.EXACT("Practicums") OR MAINSUBJECT.EXACT("Service Learning")	17544
**S4**	1 or 2 or 3	116376
**S5**	(interprofession* or "inter-profession*" or interdisciplin* or "inter-disciplin*" or interinstitut* or "inter-institut*" or interagenc* or "inter-agenc*" or intersector* or "inter-sector*" or interdepartment* or "inter-department*" or interoccupation* or "inter-occupation*" or interorgani?ation* or "inter-organi?ation*" or multiprofession* or "multi-profession*" or multidisciplin* or "multi-disciplin*" or multiinstitut* or "multi-institut*" or multiagenc* or "multi-agenc*" or multisector* or "multi-sector*" or multidepartment* or "multi-department*" or multioccupation* or "multi-occupation*" or multiorgani?ation* or "multi-organi?ation*" or transprofession* or "trans-profession*" or transdisciplin* or "trans-disciplin*" or transagenc* or "trans-agenc*" or transsector* or "trans-sector*" or transdepartment* or "trans-department*" or transoccupation* or "trans-occupation*" or transorgani?ation* or "transorgani?ation*" or "cross-training" or crossprofession* or "cross-profession*" or crossdisciplin* or "cross-disciplin*" or crossinstitut* or "cross-institut*" or crossagenc* or "cross-agenc*" or crosssector* or "cross-sector*" or crossdepartment* or "cross-department*" or crossoccupation* or "cross-occupation*" or crossorgani?ation* or "cross-organi?ation*" or team* or collaborative or IPP or IPE or ILP or IPECP)	128625

**S6**	PUB(interprofession*)	11
**S7**	MAINSUBJECT.EXACT("Interprofessional Relationship")	4134
**S8**	5 or 6 or 7	128625
**S9**	((primary or ambulatory or communit* or general or family) N/2 (care or healthcare or "health care" or ("health program" OR "health programme" OR "health programmes" OR "health programs") or ("health centre" OR "health centres") or ("health center" OR "health centers") or ("health service" OR "health services") or practice* or clinic* or practition* or medicine or doctor* or physician* or clinician*))	27052
**S10**	(("private practice" OR "private practices" OR "private practise" OR "private practitioner" OR "private practitioners") or GP or PCP or CBHW)	637
**S11**	PUB(community or "general practice*" or "general practitioner*" or "family practic*" or "family medicine" or "primary care")	14839
**S12**	MAINSUBJECT.EXACT("Community Health Services")	1526
**S13**	MAINSUBJECT.EXACT("Primary Health Care")	1363
**S14**	MAINSUBJECT.EXACT("Family Practice (Medicine)")	610
**S15**	9 or 10 or 11 or 12 or 13 or 14	41719
**S16**	(student* or learner* or resident or residents or intern or interns or PGY or graduate* or postgraduate* or "post-grad*" or undergraduate* or "under-grad*" or trainee*)	983344
**S17**	MAINSUBJECT.EXACT("Medical Students") OR MAINSUBJECT.EXACT("Nursing Students")	6164
**S18**	MAINSUBJECT.EXACT("Medical Education") OR MAINSUBJECT.EXACT("Graduate Medical Education") OR MAINSUBJECT.EXACT("Nursing Education") OR MAINSUBJECT.EXACT("Pharmaceutical Education")	17487
**S19**	16 or 17 or 18	988590
**S20**	4 and 8 and 15 and 19	702
**S21**	20 (limit to 2001- present)	568
**S22**	21 (limit to english)	568

Table 5. PsycInfo

Database: APA PsycInfo <1806 to July Week 1 2023><br>Platform: OVID<br>Date Searched: Jul 11, 2023		
#	Searches	Results
1	(clerkship* or preceptorship* or traineeship* or placement* or exposure* or practicum* or "off service" or fieldwork or "field-work" or "field training" or "field study" or "field studies" or "field education" or "field experience" or "workplace learn*" or "workplace training" or "practice	337628

	learn*" or "practice based learn*" or "practical learn*" or "experiential learn*" or "experiential education" or "work based learn*" or "work integrated learn*" or "service learning" or "deliberate practice" or rotation or rotations or "clinical practice" or "clinical environment*" or "clinical setting*" or "clinical education" or "co-op" or "cooperative education" or "collaborative learning").mp.	
2	exp experiential learning/	7408
3	cooperative education/	232
4	or/1-3 [Practicum Concept]	339206
5	(interprofession* or "inter-profession*" or interdisciplin* or "inter-disciplin*" or interinstitut* or "inter-institut*" or interagenc* or "inter-agenc*" or intersector* or "inter-sector*" or interdepartment* or "inter-department*" or interoccupation* or "inter-occupation*" or interorgani?ation* or "inter-organi?ation*" or multiprofession* or "multi-profession*" or multidisciplin* or "multi-disciplin*" or multiinstitut* or "multi-institut*" or multiagenc* or "multi-agenc*" or multisector* or "multi-sector*" or multidepartment* or "multi-department*" or multioccupation* or "multi-occupation*" or multiorgani?ation* or "multi-organi?ation*" or transprofession* or "trans-profession*" or transdisciplin* or "trans-disciplin*" or transagenc* or "trans-agenc*" or transsector* or "trans-sector*" or transdepartment* or "trans-department*" or transoccupation* or "trans-occupation*" or transorgani?ation* or "transorgani?ation*" or "cross-training" or crossprofession* or "cross-profession*" or crossdisciplin* or "cross-disciplin*" or crossinstitut* or "cross-institut*" or crossagenc* or "cross-agenc*" or crosssector* or "cross-sector*" or crossdepartment* or "cross-department*" or crossoccupation* or "cross-occupation*" or crossorgani?ation* or "cross-organi?ation*" or team* or collaborative or IPP or IPE or ILP or IPECP).mp.	202711
6	interprofession*.jx.	1726
7	or/5-6 [Interprofessional Concept]	202803
8	((primary or ambulatory or communit* or general or family) adj2 (care or healthcare or "health care" or "health program*" or "health centre*" or "health center*" or "health service*" or practice* or clinic* or practition* or medicine or doctor* or physician* or clinician*)).mp.	134060
9	("private pract*" or GP or PCP or CBHW).mp.	12698
10	(community or "general practice*" or "general practitioner*" or "family practic*" or "family medicine" or "primary care").jx.	32071
11	community services/	18154
12	primary health care/	21252
13	Private Practice/	1599
14	Family Physicians/	1632
15	General Practitioners/	6367
16	or/8-15 [Primary Care Concept]	177738

17	(student* or learner* or resident or residents or intern or interns or PGY or graduate* or postgraduate* or "post-grad*" or undergraduate* or "under-grad*" or trainee*).mp.	863032
18	medical residency/ or medical students/ or medical education/ or medical internship/ or dental education/ or dental students/ or nursing education/ or nursing students/	42394
19	or/17-18 [Student Concept]	870454
20	and/4,7,16,19 [FINAL set; no limits]	864
21	limit 20 to (english language and yr="2001 -Current") [FINAL set; with limits applied]	746

Table 6. Scopus

Database: Scopus<br>Platform:<br>Date Searched: Jul 11, 2023		
#	Searches	Results
1	TITLE-ABS-KEY (clerkship* OR preceptorship* OR traineeship* OR placement* OR exposure* OR practicum* OR "off service" OR fieldwork OR "field-work" OR "field training" OR "field study" OR "field studies" OR "field education" OR "field experience" OR "workplace learn*" OR "workplace training" OR "practice learn*" OR "practice based learn*" OR "practical learn*" OR "experiential learn*" OR "experiential education" OR "work based learn*" OR "work integrated learn*" OR "service learning" OR "deliberate practice" OR rotation OR rotations OR "clinical practice" OR "clinical environment*" OR "clinical setting*" OR "clinical education" OR "co-op" OR "cooperative education" OR "collaborative learning")	3,548,696
2	TITLE-ABS-KEY (interprofession* OR "inter-profession*" OR interdisciplin* OR "inter-disciplin*" OR interinstitut* OR "inter-institut*" OR interagenc* OR "inter-agenc*" OR intersector* OR "inter-sector*" OR interdepartment* OR "inter-department*" OR interoccupation* OR "inter-occupation*" OR interorgani?ation* OR "inter-organi?ation*" OR multiprofession* OR "multi-profession*" OR multidisciplin* OR "multi-disciplin*" OR multiinstitut* OR "multi-institut*" OR multiagenc* OR "multi-agenc*" OR multisector* OR "multi-sector*" OR multidepartment* OR "multi-department*" OR multioccupation* OR "multi-occupation*" OR multiorgani?ation* OR "multi-organi?ation*" OR transprofession* OR "trans-profession*" OR transdisciplin* OR "trans-disciplin*" OR transagenc* OR "trans-agenc*" OR transsector* OR "trans-sector*" OR transdepartment* OR "trans-department*" OR transoccupation* OR "trans-occupation*" OR transorgani?ation* OR "transorgani?ation*" OR "cross-training" OR crossprofession* OR "cross-profession*" OR crossdisciplin* OR "cross-	1,364,615

	disciplin*" OR crossinstitut* OR "cross-institut*" OR crossagenc* OR "cross-agenc*" OR crosssector* OR "cross-sector*" OR crossdepartment* OR "cross-department*" OR crossoccupation* OR "cross-occupation*" OR crossorgani?ation* OR "cross-organi?ation*" OR team* OR collaborative OR ipp OR ipe OR ilp OR ipecp)	
3	SRCTITLE (interprofession*)	3,831
4	2 or 3	1,365,211
5	TITLE-ABS-KEY (((primary OR ambulatory OR communit* OR general OR family) W/2 (care OR healthcare OR "health care" OR "health program*" OR "health centre*" OR "health center*" OR "health service*" OR practice* OR clinic* OR practition* OR medicine OR doctor* OR physician* OR clinician*)))	845,825
6	TITLE-ABS-KEY ("private pract*" OR gp OR pcp OR cbhw)	151,645
7	SRCTITLE (community OR "general practice*" OR "general practitioner*" OR "family practic*" OR "family medicine" OR "primary care")	199,783
8	5 or 6 or 7	1,104,683
9	TITLE-ABS-KEY (student* OR learner* OR resident OR residents OR intern OR interns OR pgy OR graduate* OR postgraduate* OR "post-grad*" OR undergraduate* OR "under-grad*" OR trainee*)	2,166,925
10	1 and 4 and 8 and 9	3,146
11	10 (limited to 2001 to present)	2,783
12	11 (limited to english)	2,707

Table 7. Social Services Abstracts

Database: Social Services Abstracts<br>Platform: ProQuest<br>Date Searched: Jul 11, 2023		
#	Searches	Results
S1	NOFT(clerkship* or preceptorship* or traineeship* or placement* or exposure* or practicum* or "off service" or fieldwork or "field-work" or "field training" or "field study" or "field studies" or "field education" or "field experience" or ("workplace learning") or "workplace training" or ("practice learning") or "practice based learn*" or ("practical learning") or ("experiential learning") or "experiential education" or "work based learn*" or "work integrated learn*" or "service learning" or "deliberate practice" or rotation or rotations or "clinical practice" or ("clinical environment" OR "clinical environments") or ("clinical setting" OR "clinical settings") or "clinical education" or "co-op" or "cooperative education" or "collaborative learning")	29244
S2	MAINSUBJECT.EXACT("Field Instruction")	256

S3	MAINSUBJECT.EXACT("Service Learning") OR MAINSUBJECT.EXACT("Internship Programs")	333
S4	1 or 2 or 3	29344
S5	NOFT(interprofession* or "inter-profession*" or interdisciplin* or "inter-disciplin*" or interinstitut* or "inter-institut*" or interagenc* or "inter-agenc*" or intersector* or "inter-sector*" or interdepartment* or "inter-department*" or interoccupation* or "inter-occupation*" or interorgani?ation* or "inter-organi?ation*" or multiprofession* or "multi-profession*" or multidisciplin* or "multi-disciplin*" or multiinstitut* or "multi-institut*" or multiagenc* or "multi-agenc*" or multisector* or "multi-sector*" or multidepartment* or "multi-department*" or multioccupation* or "multi-occupation*" or multiorgani?ation* or "multi-organi?ation*" or transprofession* or "trans-profession*" or transdisciplin* or "trans-disciplin*" or transagenc* or "trans-agenc*" or transsector* or "trans-sector*" or transdepartment* or "trans-department*" or transoccupation* or "trans-occupation*" or transorgani?ation* or "transorgani?ation*" or "cross-training" or crossprofession* or "cross-profession*" or crossdisciplin* or "cross-disciplin*" or crossinstitut* or "cross-institut*" or crossagenc* or "cross-agenc*" or crosssector* or "cross-sector*" or crossdepartment* or "cross-department*" or crossoccupation* or "cross-occupation*" or crossorgani?ation* or "cross-organi?ation*" or team* or collaborative or IPP or IPE or ILP or IPECP)	24597
S6	PUB(interprofession*)	295
S7	MAINSUBJECT.EXACT("Interprofessional Approach")	255
S8	MAINSUBJECT.EXACT("Interdisciplinary Approach")	914
S9	5 or 6 or 7 or 8	24597
S10	NOFT((primary or ambulatory or communit* or general or family) N/2 (care or healthcare or "health care" or ("health program" OR "health programme" OR "health programmes" OR "health programs") or ("health centre" OR "health centres") or ("health center" OR "health centers") or ("health service" OR "health services") or practice* or clinic* or practition* or medicine or doctor* or physician* or clinician*))	35867
S11	NOFT("private pract*" or GP or PCP or CBHW)	1262
S12	PUB(community or "general practice*" or "general practitioner*" or "family practic*" or "family medicine" or "primary care")	17757
S13	MAINSUBJECT.EXACT("Primary Health Care")	1341
S14	MAINSUBJECT.EXACT("Private Practice")	114
S15	10 or 11 or 12 or 13 or 14	47301
S16	NOFT(student* or learner* or resident or residents or intern or interns or PGY or graduate* or postgraduate* or "post-grad*" or undergraduate* or "under-grad*" or trainee*)	52565
S17	MAINSUBJECT.EXACT("Medical Students")	275
S18	16 or 17	52565

S19	4 and 9 and 15 and 18	121
S20	19 [limited to 2001 - present]	101
S21	21 [limited to english]	101
