# Supplementary material for: Exploring experiential learning within interprofessional practice education initiatives for pre-licensure healthcare students: a scoping review
Source: BMC Med Educ. 2024 Feb 13;24:139. doi: 10.1186/s12909-024-05114-w (PMC10863283; doi:10.1186/s12909-024-05114-w)
Supplement: Supplementary file 2 — Additional file 2. Data Points for Extraction. [file 12909_2024_5114_MOESM2_ESM.pdf]

Supplementary File #2

Data Points for Extraction

Data from articles for this scoping review were extracted in pairs by two members of our research team and entered into an Excel spreadsheet for collation and analysis. The categories and subcategories for data extraction were first developed by the project team leads (DAN, JLP, and GH), pilot tested, and refined as the project evolved. The categories and subcategories are as follows:

A. Descriptive Information of Article

- Authors
- Year of publication
- Country of origin
- Title
- Article type
- Methodological approach (if applicable)
- Study design (if applicable)
- Purpose(s) of Article (if stated)
- Objectives (if stated)
- Research question(s) (if applicable)

B. Descriptive Information of IPP Education Initiative

- Taxonomy of education initiative
- Student disciplines involved
- Student level in program
- Nature of student involvement
- Healthcare setting for initiative
- Target population of service provided
- Type of service/care provided by students
- Framework or theory for IPP (if stated)
- Description of IPP education initiative/strategy (if stated)
- Intention of IPP education/strategy (if stated)
- Faculty/professional involvement
- Other stakeholders involved

C. Teaching and Learning Strategies

- Prerequisite/corequisite courses or required preparation
- Specific educational strategies/activities/approaches
- Duration of education opportunity
- Expectations/roles of learners

D. Evaluation of IPP Initiative

- Outcomes evaluated
- Evaluation method(s) used
- Findings
- Author stated limitations

E. Author conclusions/recommendations if applicable

F. Research team reviewer observations
