# Supplementary material for: Exploring experiential learning within interprofessional practice education initiatives for pre-licensure healthcare students: a scoping review
Source: BMC Med Educ. 2024 Feb 13;24:139. doi: 10.1186/s12909-024-05114-w (PMC10863283; doi:10.1186/s12909-024-05114-w)
Supplement: Supplementary file 3 — Additional file 3. Articles Included for Extraction from Literature Search of Interprofessional Practice (IPP) Education Initiatives. [file 12909_2024_5114_MOESM3_ESM.pdf]

Supplementary File #3

Articles Included for Extraction from Literature Search of Interprofessional Practice (IPP) Education Initiatives

**Author**	**Country**	**Literature Typology** (methodological approach, if applicable	**Purpose or Aim of Article**	***Setting*(s)** **IPP Education Approach/ Strategies**	**Student Disciplines Involved**
Adams, A.L., Cadle, R. M., Amspoker, A. B., Martin, L. A., Wilmer, S. R., Fernandez, J. M., Pereira, C. E., Shah, R. M., Woodard, L. D., & Hatfield, C. L. (2019). Preliminary evaluation of pharmacy resident integration within an academic interprofessional patient aligned care team at a Veterans Affairs Academic Medical Center. *Journal of Interprofessional Education and Practice, 15*(), 55-59.<br>DOI: 10.1016/j.xjep.2019.02.003	United States	Primary research<br>(Mixed methods)	The purpose of this paper was to describe pharmacy residents' experience and other post-graduate trainees' perspectives of pharmacy involvement in the initial stages of an interprofessional ambulatory care training program.	*CarePlus Clinic and Ambulatory Care*<br>- didactic sessions on interprofessional and clinical topics led by faculty<br>- reflections at the end of sessons<br>- collaborative work on evaluation of medication use, quality improvement projects, and other evaluation activities<br>- 1-hour case presentations by trainees to all members and faculty	Medicine<br>Nurse practitioners<br>Pharmacy<br>Psychology
Aggar, C., Mozolic-Staunton, B., Lovi, R. J., Scorey, M., Kemp, M., Walker, T., & Lewis, S. (2020). An interprofessional clinical placement in a primary healthcare setting: A pilot study. *Internet Journal of Allied*	Australia	Primary research<br>(quantitative – longitudinal pilot study)	The aim of this study was to evaluate the impact of IPE clinical placement in a primary healthcare setting on students' perceptions of interprofessional education.	*Primary care*<br>- clinical placements<br>- daily meetings and debriefs	Nursing<br>Occupational therapy<br>Podiatry<br>Speech pathology

Author	Country	Literature Typology (methodological approach, if applicable	Purpose or Aim of Article	*Setting*(s) IPP Education Approach/ Strategies	Student Disciplines Involved
Health Sciences and Practice, *18*(1), 6.<br>DOI: 10.46743/1540-580X/2020.1844.				- development of health promotion initiative	
Anderson, E. S., Ford, J., & Kinnair, D. J. (2016). Interprofessional education and practice guide no. 6: developing practice-based interprofessional learning using a short placement model. *Journal of Interprofessional Care*, *30*(4), 433-440.<br>DOI: 10.3109/13561820.2016.1160040	United Kingdom	Practice guide	This guide offers key lessons developed over many years for setting up practice-based IPE. The learning model has been adapted and adopted in different settings and countries and offers a method for engaging clinical frontline practitioners in learning with, and from learners who can help support and in some instances advance care delivery.	Not applicable	None specified (multiple disciplines described in curriculum mapping)
Anderson, E. S., & Lennox, A. (2009). The Leicester model of interprofessional education: developing, delivering and learning from student voices for 10 years. *Journal of Interprofessional Care, 23*(6), 557-573.<br>DOI: 10.3109/13561820903051451	United Kingdom	Program description<br>(Mixed methods)	An aim/purpose of article was not explicity stated. Authors described the original setting and presents the evaluation outcomes of the Leicester Model's ''Health in the Community'' course that is delivered in city-centre communities. It traces a ten-year trajectory of IP student group evaluations that helped shape this learning experience.	*Health and social care centre*<br>- small groups of mixed disciplines<br>- joint interview of clients in home visit<br>- interviews with healthcare practitioners	Medicine<br>Nursing<br>Pharmacy<br>Social work<br>Speech & language therapy
Anderson, E. S., Smith, R., & Thorpe, L. N. (2010). Learning from lives together: medical and social work	United Kingdom	Primary research<br>(Mixed methods)	The study aimed to evaluate an interprofessional community-	*Community hospitals*	Medicine<br>Social work

Author	Country	Literature Typology (methodological approach, if applicable	Purpose or Aim of Article	*Setting*(s) IPP Education Approach/ Strategies	Student Disciplines Involved
students' experiences of learning from people with disabilities in the community. *Health and Social Care in the Community, 18*(3), 229-240. DOI: 10.1111/j.1365-2524.2010.00921.x			based learning event, focussing on disability.	- small IP groups of 3-4 students to work with clients with disabilities<br>- reflective evaluation	
Anderson, E. S., & Thorpe, L. N. (2014). Students improve patient care and prepare for professional practice: an interprofessional community-based study. *Medical Teacher, 36*(6), 495-504. DOI: 10.3109/0142159X.2014.890703	United Kingdom	Primary research (Qualitative – exploratory qualitative study, realist approach)	An aim/purpose of article was not explicity stated. Authors prospectively followed interprofessional student teams in mid-training using an IPL model to assess how learning contributions to patient and service outcomes where the model is used in inner city primary care.	*Inner city primary care teams*<br>- small IP groups of 3-5 students work with professionals<br>- written feedback	Medicine<br>Nursing<br>Social work<br>Pharmacy<br>Speech and language therapy
Block, L., LaVine, N. A., Martinez, J., Strawser, J., Lu, C., Cacace, F., Fornari, A., Conigliaro, J., & Coletti, D. J. (2021). A novel longitudinal interprofessional ambulatory training practice: the improving patient access care and cost through training (IMPACcT) clinic. *Journal of Interprofessional Care, 35*(3), 472-475. DOI: 10.1080/13561820.2020.1751595	United States	Program evaluation (Quantitative)	Evaluation of learner outcomes, provider continuity, quality of clinical outcomes, and patient satisfaction of the IMPACcT training clinic.	*Primary care facilities*<br>- brief huddles to collaborate, plan workflows, discuss topics and negotiate approaches to patient care<br>- interprofessional signout	Medicine<br>Physician<br>Physician assistant<br>Pharmacy<br>Psychology trainees

**Author**	**Country**	**Literature Typology** (methodological approach, if applicable	**Purpose or Aim of Article**	***Setting*(s) IPP Education Approach/ Strategies**	**Student Disciplines Involved**
Bluestein, D., & Cubic, B. A. (2009). Psychologists and primary care physicians: a training model for creating collaborative relationships. *Journal of Clinical Psychology in Medical Settings, 16*(1), 101-112.<br>DOI: 10.1007/s10880-009-9156-9	United States	Program description	This article described the importance of primary care and psychology partnering to create integrated care models and makes the case that such partnerships are not only beneficial to patients but to both professions.	*Primary care*<br>- training in interdisciplinary team skills<br>- additional didactics on psychosocial issues and multicultural diversity<br>- collaboratively designed treatments	Medical residents<br>Psychology interns
Bondevik, G. T., Holst, L., Haugland, M. J., Baerheim, A., & Raaheim, A. (2015). Interprofessional workplace learning in primary care: students from different health professions work in teams in real-life settings. *International Journal of Teaching and Learning in Higher Education, 27*(2), 175-182.<br>DOI: no doi	Norway	Primary research<br>(Qualitative)	The aim of this study is to describe and discuss the self-reported experience from Norwegian health care students participating in interprofessional workplace learning in primary care.	*Nursing homes and public health clinics*<br>- interview and assess patients<br>- collaboration in writing an individualized treatment plan<br>- work in groups of 4-5	Dental hygiene<br>Medicine<br>Midwifery<br>Nursing (public health)<br>Nutrition<br>Pharmacy<br>Physiotherapy
Brewer, M. L., Flavell, H. L., & Jordan, J. (2017). Interprofessional team-based placements: the importance of space, place, and facilitation. *Journal of Interprofessional Care, 31*(4), 429-437.	Australia	Primary research<br>(Case study – exploratory)	This study aimed to understand students' perceptions of the impact of an interprofessional placement on their learning.	*Primary schools and aged care facility*<br>- collaborated at different points to provide interventions	Counselling<br>Dietetics<br>Nursing<br>Occupational therapists

**Author**	**Country**	**Literature Typology** (methodological approach, if applicable	**Purpose or Aim of Article**	***Setting***(s) **IPP Education Approach/ Strategies**	**Student Disciplines Involved**
DOI: 10.1080/13561820.2017.1308318				tailored to professions.	Pharmacy<br>Physiotherapy<br>Psychology<br>Speech pathology
Bridges, D. R., Davidson, R. A., Odegard, P. S., Maki, I. V., & Tomkowiak, J. (2011). Interprofessional collaboration: three best practice models of interprofessional education. *Medical Education Online, 16*(1), 6035.<br>DOI: 10.3402/meo.v16i0.6035	United States	Program description	An aim/purpose of article was not explicity stated. Authors showcased three exemplary models of collaborative and interprofessional educational experiences so that other institutions may benefit from these when creating interprofessional curricula.	*Placement at clinical site; Home visits to families; Rural and under-served populations*<br>Varied according to institution, but included:<br>- observation of clients and discussion of cases<br>- joint meetings with course coordinators<br>- post-interview reflection with mentor<br>- IP teams of 3 students for visits<br>- "windshield survey" of client's neighborhood<br>- service projects	Clinical laboratory<br>Dentistry<br>Medicine<br>Nurse anesthetists<br>Nursing<br>Nutrition<br>Pharmacy<br>Physical therapy<br>Physician assistants<br>Podiatry<br>Psychology<br>Public health<br>Social work

**Author**	**Country**	**Literature Typology** (methodological approach, if applicable	**Purpose or Aim of Article**	***Setting*(s)** **IPP Education Approach/ Strategies**	**Student Disciplines Involved**
Buff, S. M., Jenkins, K., Kern, D., Worrall, C., Howell, D., Martin, K., Brown, D., White, A., & Blue, A. (2015). Interprofessional service-learning in a community setting: findings from a pilot study. *Journal of Interprofessional Care, 29*(2), 159-161.<br>DOI: 10.3109/13561820.2014.934956	United States	Primary research<br>(Mixed methods)	The aim of this study was to share the results from an interprofessional service-learning project (ISLP) targeting students across eight disciplines.	*Community site*<br>- outreach activities<br>- appreciating interprofessional collaboration	Biomedical science<br>Dietetic interns<br>Masters in health administration<br>Medicine<br>Nursing<br>Pharmacy<br>Physical therapy<br>Physician assistant
Busen, N. H. (2014). An interprofessional education project to address the health care needs of women transitioning from prison to community reentry. *Journal of Professional Nursing, 30*(4), 357-366.<br>DOI: 10.1016/j.profnurs.2014.01.002	United States	Program description	The purpose of this article was to describe the development of an IPE course that illustrates the advantages and challenges associated with the process.	*University health science center and community NGO site*<br>- interactive teaching-learning sessions<br>- worked in teams<br>- develop a curriculum<br>- collaborated with community partners<br>- step back exercise and reflective journaling	Dentistry<br>Nursing<br>Nutrition

**Author**	**Country**	**Literature Typology** (methodological approach, if applicable	**Purpose or Aim of Article**	***Setting*(s)** **IPP Education Approach/ Strategies**	**Student Disciplines Involved**
Cappiello, J. D., Joy, J., Smith, P., & Orgren, R. A. (2015). The SEARCH project: Acquainting students in the health professions with interprofessional care. *Journal of Allied Health, 44*(2), 91-95.<br>DOI: no doi	United States	Primary research<br>(Qualitative)	The purpose of this paper was to provide affirmative evidence for an interprofessional education and practice project that relies upon shared responsibility for quality outcomes.	*Post-secondary educational institutions*<br>- shadowed HCPs<br>- presentations, team building exercises<br>- team conferences<br>- debriefings	Medicine<br>Nursing<br>Physician assistant
Chen, P., Clark, M., Sharma, M., Troya, Y., Cenzer, I., & Rivera, J. (2021). A longitudinal workplace-based interprofessional curriculum for graduate learners in a geriatrics patient-centered medical home. *Journal of Interprofessional Education and Practice, 24*, 100459.<br>DOI: 10.1016/j.xjep.2021.100459	United States	Program evaluation	An aim/purpose of article was not explicity stated. Authors described a year-long workplace-based IP curriculum that incorporated workplace learning principles to improve IP collaborative competencies, improve perceived teamness, and provide team-based care to older adults.	*Academic patient-centered medical home*<br>- active individual and collective provision of care<br>- engagement in operations of the practice	Law (medical-legal focus)<br>Medicine<br>Pharmacy<br>Social work
Chirico, M., Thompson, J. R., & Steil, C. (2014). Development and implementation of a collaborative interprofessional learning program. *Currents in Pharmacy Teaching and Learning, 6*(4), 550-557.<br>DOI: 10.1016/j.cptl.2014.04.006	United States	Program description<br>(Quantitative)	This article described the implementation and first two years of follow-up of a novel interprofessional program.	*Post-secondary educational institutions*<br>- weekly reflection<br>- worked in teams of 4<br>- co-curricular materials	Medicine<br>Nurse practitioners<br>Pharmacy<br>Social work

**Author**	**Country**	**Literature Typology** (methodological approach, if applicable	**Purpose or Aim of Article**	***Setting*(s)** **IPP Education Approach/ Strategies**	**Student Disciplines Involved**
				- self-directed learning<br>- active learning strategy "debrief"	
Chrisman-Khawam, L., Abdullah, N., & Dhoopar, A. (2017). Teaching health-care trainees empathy and homelessness IQ through service learning, reflective practice, and altruistic attribution. *The International Journal of Psychiatry in Medicine, 52*(3), 245-254.<br>DOI: 10.1177/0091217417730288	United States	Program description	This article described a novel interprofessional curriculum designed to address the needs of homeless patients in a Midwestern of the United States which has high rates of poverty.	*Homeless shelters and health care centers*<br>- working in IP groups<br>- post-session debrief	Clinical psychology<br>Medicine<br>Nursing<br>Pharmacy<br>Psychiatry<br>Social work<br>Undergraduate pre-medical program
Claeys, T., Dolmans, D., & de Nooijer, J. (2022). Design and evaluation of a team-based interprofessional practice placement: A design-based research approach. *Medical Teacher, 44*(8), 866-871.<br>DOI: 10.1080/0142159X.2022.2041188	Netherlands	Primary research (Qualitative – design-based research)	The study explored the design and evaluation of Team-based Interprofessional Practice Placements to support undergraduate students in gaining a better understanding of the complexity of patient problems in primary care settings and of collaboration within interprofessional teams.	*Project organized in the community*<br>- work in small IP teams with clients and caregivers<br>- support/coaching from teachers using a scaffold approach to learning	Dietetics<br>Nursing<br>Occupational therapy<br>Social work<br>Speech therapy
Claiborne, D. M., Poston, R., & Joufi, A. (2020). Innovative collaborative service-learning experience among	United States	Primary research (Quantitative)	The purpose of this study was to pilot an innovative, collaborative service-learning (ICSL)	*Child development center*	Dental hygiene<br>Nurse practitioner

**Author**	**Country**	**Literature Typology** (methodological approach, if applicable	**Purpose or Aim of Article**	***Setting*(s)** **IPP Education Approach/ Strategies**	**Student Disciplines Involved**
dental hygiene and nurse practitioner students: A pediatric oral health pilot study. *The Journal of Dental Hygiene, 94*(3), 29-36.<br>DOI: no doi			experience for dental hygiene and primary care nurse practitioner students to address pediatric oral health.	- collaboration on development of education materials<br>- hands-on learning and activities	
Copley, J. A., Allison, H. D., Hill, A. E., Moran, M. C., Tait, J. A., & Day, T. (2007). Making interprofessional education real: a university clinic model. *Australian Health Review, 31*(3), 351-357.<br>DOI: 10.1071/ah070351	Australia	Program description	The purpose of this paper is to report on the development of university-conducted clinical services that provide students with the opportunity for authentic IP clinical education.	*University-conducted clinics*<br>- collaborative client goal setting<br>- weekly therapy session planning	Music therapy<br>Occupational therapy<br>Speech pathology
Cox, L., Fugit, A. M., Ryan, M., Gregory, A., & Mayer, S. (2014). Una Vida Sana!: An interprofessional service-learning outreach project in an underserved Latino population. *Currents in Pharmacy Teaching and Learning, 6*(4), 577-584.<br>DOI: 10.1016/j.cptl.2014.03.002	United States	Program description	An aim/purpose of article was not explicity stated. Authors described three objectives related to how an IP service-learning projected was developed, a description of community participants involved in the project's activies, and report on student perceptions of preparing for and engaging in the project.	*Large free clinic (CrossOver Healthcare Ministry)*<br>- active participation in project leadership (e.g. recruitment of volunteers)<br>- coordination of IP training for students	Medicine<br>Nursing<br>Pharmacy
Croker, A., Fisher, K., & Smith, T. (2015). When students from different professions are co-located: the importance of interprofessional rapport for learning to work together.	Australia	Primary research<br>(Qualitative – exploratory study	The study aimed to explore the issue of co-location and its effects on how students learn to work with other professions.	*Community health services*<br>- shared accommodations	Nursing<br>Nutrition and dietetics<br>Medicine

**Author**	**Country**	**Literature Typology** (methodological approach, if applicable	**Purpose or Aim of Article**	***Setting*(s)** **IPP Education Approach/ Strategies**	**Student Disciplines Involved**
Journal of Interprofessional Care, 29(1), 41-48.<br>DOI: 10.3109/13561820.2014.937481		using hermeneutics)		- IPL sessions	Pharmacy<br>Speech pathology
Danhausen, K., Joshi, D., Quirk, S., Miller, R., Fowler, M., & Schorn, M. N. (2015). Facilitating access to prenatal care through an interprofessional student-run free clinic. *Journal of Midwifery & Women's Health, 60*(3), 267-273.<br>DOI: 10.1111/jmwh.12304	United States	Primary research<br>(Quantitative – retrospective chart review)	The purpose of this article was to present the model of a prenatal clinic offered by a student-run health center.	*Primary care*<br>- team of 2<br>- provision of patient care (learning with and from each other)	Medicine<br>Midwifery
Davis, L. I., Wright, D. J., Gutierrez, M. S., Nam, J. J., Nguyen, J., & Waite, A. T. (2015). Interprofessional global service learning: a pharmacy and nursing practice experience in Botswana. *Currents in Pharmacy Teaching and Learning, 7*(2), 169-178.<br>DOI: 10.1016/j.cptl.2014.11.017	United States	Program description<br>(Mixed methods)	This paper aimed to describe the development, implementation and assessment of an interprofessional global service-learning experience in rural government-run clinics and community hospital for pharmacy and nursing students in Kanye, Botswana.	*Primary care*<br>- engagement in clinical practice	Nursing<br>Pharmacy
Dolce, M. C., Parker, J. L., Marshall, C., Riedy, C. A., Simon, L. E., Barrow, J., Ramos, C. R., & DaSilva, J. D. (2017). Expanding collaborative boundaries in nursing education and practice: the nurse practitioner-dentist	United States	Program description	The purpose of this paper was to describe the design and implementation of a novel interprofessional collaborative practice education program, the Nurse Practitioner-Dentist Model for Primary Care (NPD Program). The NPD Program	*University dental facility*<br>- self-directed online modules<br>- IPC practice leadership and teamwork training	Dentistry<br>Nurse practitioner

Author	Country	Literature Typology (methodological approach, if applicable	Purpose or Aim of Article	*Setting*(s) IPP Education Approach/ Strategies	Student Disciplines Involved
model for primary care. *Journal of Professional Nursing, 33*(6), 405-409. DOI: 10.1016/j.profnurs.2017.04.002			expands collaborative boundaries in nursing and dental education by integrating primary care services, provided by a nurse practitioner, within an academic dental practice.	- supervised clinical rotations<br>- constructive feedback for team and individual	
Doucet, J. R., Fournier, T. A., Bishop, C. M., Gaudet, D. J., & Nagel, D. A. (2021). Pilot of a community-based interprofessional "student-infused" pulmonary rehabilitation program in Saint John, New Brunswick. *Canadian Journal of Respiratory Therapy, 57*, 26-31. DOI: 10.29390/cjrt-2020-053	Canada	Primary research (Quantitative – pre and post test quasi-experimental)	The overall goal of this project was to pilot implementation of a community-based interprofessional "student-infused" PR program and examine the efficacy of such an initiative.	*Post-secondary educational institution*<br>- working in IP team	Nursing<br>Practical nursing<br>Pharmacy technician<br>Respiratory therapy
Elliott, K. J., Annear, M. J., Bell, E. J., Palmer, A. J., & Robinson, A. L. (2015). Residents with mild cognitive decline and family members report health students 'enhance capacity of care' and bring 'a new breath of life' in two aged care facilities in Tasmania. *Health Expectations, 18*(6), 1927-1940. DOI: 10.1111/hex.12236	Australia	Primary research (Mixed methods)	The purpose of the article was to describe the perspective of a select group of residents with mild cognitive decline and their family members on student doctors and nurses.	*Aged-care facilities*<br>- collaborative assessment and recommendations	Medicine<br>Nursing
Flinn, S., Kloos, A., Teaford, M., Clark, K., & Szucs, K. (2009). Helping hands for healthy living: a	United States	Program evaluation	The purpose of this article was to describe the development of a unique interdisciplinary service-	*Primary care*	Physical therapy

Author	Country	Literature Typology (methodological approach, if applicable	Purpose or Aim of Article	*Setting*(s) IPP Education Approach/ Strategies	Student Disciplines Involved
collaborative service learning project with occupational and physical therapy students. *Occupational Therapy in Health Care, 23*(2), 146-167.<br>DOI: 10.1080/07380570902779807		(Mixed methods)	learning course and to evaluate the impact of this experience on allied health students' professional development.	- one 4-hour classroom experience<br>- four 2-hour service activities	Occupational therapy
Forbes, R., Beckman, E., Tower, M., Mandrusiak, A., Mitchell, L. K., Sexton, C. T., Cunningham, B., & Lewis, P. A. (2021). Interprofessional, student-led community health clinic: expanding service provision and clinical education capacity. *Australian Health Review, 45*(2), 255-260.<br>DOI: 10.1071/AH20021	Australia	Primary research (Case study)	The purpose of this article was to describe the operation of a new interprofessional student-led community health model over its initial 13-month period of operation.	*Post-secondary educational institution*<br>- daily team meetings<br>- case conferences and informal student discussions<br>- collaborative consultations/assess ments<br>- frequent debriefs	Counselling<br>Dietetics<br>Exercise physiology<br>Nursing<br>Occupational therapy<br>Physiotherapy<br>Psychology<br>Social work
Frakes, K., Brownie, S., Davies, L., Thomas, J., Miller, M., & Tyack, Z. (2014). Experiences from an interprofessional student-assisted chronic disease clinic. *Journal of Interprofessional Care, 28*(6), 573-575.<br>DOI: 10.3109/13561820.2014.917404	Australia	Primary research (Mixed methods)	The purpose of this article was to provide early evaluative information concerning student experiences included in self-reported changes in practice.	*Allied health partnership*<br>- IP team with professional specific group work<br>- case conferences<br>- reflective practice	Exercise physiology<br>Nutrition and dietetics<br>Occupational therapy<br>Pharmacy<br>Podiatry

Author	Country	Literature Typology (methodological approach, if applicable	Purpose or Aim of Article	*Setting*(s) IPP Education Approach/ Strategies	Student Disciplines Involved
					Social work
Fries, K. S., Bowers, D. M., Gross, M., & Frost, L. (2013). Service learning in Guatemala: using qualitative content analysis to explore an interdisciplinary learning experience among students in health care professional programs. *Journal of Multidisciplinary Healthcare, 6*, 45-52.<br>DOI: 10.2147/JMDH.S35867	United States	Primary research<br>(Qualitative – inductive approach; content analysis)	The aim of this article was to describe the experiences of students in health care professional programs from nursing, occupational, and physical therapy disciplines as they collaborated to provide health care to marginalized Guatemalan citizens over a 7-day period.	*Community and primary care*<br>- debrief sessions<br>- provide supervised health care in IP triad groups<br>- reflective learning through journaling	Nursing<br>Occupational therapy<br>Physical therapy
Garcia, J. D., & Frank, R. A. (2016). Nontraditional approach for interprofessional service learning in a doctor of physical therapy program: The Farm Worker Family Health Program. *Frontiers in Communication, 1*.<br>DOI: 10.3389/fcomm.2016.00012	United States	Program description	The purpose of the article was to describe the program and identify aspeccts of that may be impactful for enhancing skills of communication, cultural competency, and interprofessional collaboration for student physical therapists.	*Farm Worker Family Health Program (rural)*<br>- work in IP groups to assess and screen patients<br>- use case studies to problem solve with peers<br>- engage in group activities and personal reflection	Nursing<br>Pharmacy<br>Physical therapy<br>Psychology<br>Public health
Gentry, C., Espiritu, E., Schorn, M. N., Hallmark, B., Bryan, M., Prather, P., Villalta-Gill, V., Offodile, R., & Wilkins, C. (2021). Engaging the	United States	Primary research<br>(Mixed methods)	This article described a longitudinal collaborative interinstitutional IPE project that included students from five	*Not applicable*<br>- didactic sessions	Dentistry<br>Medicine<br>Nursing

**Author**	**Country**	**Literature Typology** (methodological approach, if applicable	**Purpose or Aim of Article**	***Setting*(s) IPP Education Approach/ Strategies**	**Student Disciplines Involved**
community through a longitudinal, interprofessional, interinstitutional experiential learning collaboration. *Currents in Pharmacy Teaching and Learning, 13*(2), 169-176.<br>DOI: 10.1016/j.cptl.2020.09.012			institution and ten programs, in engaging community partners (CP) while delivering core IPE competencies.	- project planning and implementation sessions<br>- presentation to community partner	Nutrition<br>Occupational therapy<br>Pharmacy<br>Physical therapy<br>Public health<br>Social work<br>Speech & hearing program
Gordon, S., Lind, C., Hall, K., & Baker, N. (2021). Attaining and assessing the Australian interprofessional learning competencies. *Journal of Interprofessional Care, 35*(2), 301-309.<br>DOI: 10.1080/13561820.2020.1712335	Australia	Primary research (Qualitative)	This paper presented the qualitative analysis of a mixed method study that evaluates students' experiences of the IPLCs during 'Inspiring Health,' a community-based interprofessional placement, and determines whether reflections can effectively assess IPLC attainment.	*Community collaborative program*<br>- full medical history assessment<br>- reflective writing	Audiology<br>Clinical science<br>Health science<br>Nursing<br>Physiotherapy<br>Social work<br>Speech pathology
Gruss, V., & Hasnain, M. (2021). Building the future geriatrics workforce through transformative interprofessional education and community-engaged experiential learning. *Journal of Interprofessional Education & Practice, 22*, 100389 .<br>DOI: 10.1016/j.xjep.2020.100389	United States	Program evaluation	This paper describes the course structure, process and program evaluation findings from the initial years of implementation.	*Senior Center, Sub-Acute in-patient Rehabilitation Hospital, House Calls program and Hospice and Palliative Care Center.*	

Author	Country	**Literature Typology** (methodological approach, if applicable	Purpose or Aim of Article	***Setting*(s)** IPP Education Approach/ Strategies	Student Disciplines Involved
				- orientation to program<br>- open discussions, discharge planning, end-of-life debate<br>- assessments, home visits, and team care planning<br>- reflection exercises	
Gudmundsen, A. C., Norbye, B., Abrandt Dahlgren, M., & Obstfelder, A. (2018). Interprofessional student meetings in municipal health service – Mutual learning towards a community of practice in patient care. *Journal of Interprofessional Care, 33*(1), 93-101.<br>DOI: 10.1080/13561820.2018.1515732	Norway	Primary research<br>(Qualitative – ethnography)	This paper investigated how mutual engagement evolves in interprofessional student meetings when medical, nursing, occupational therapy and physiotherapy students shape their own collaboration and learning in patient care.	*Geriatric rehab, nursing homes and community health services in rural settings*<br>- IP collaboration in patient care<br>- assigned in groups responsibility to deliver relevant health services<br>- participate in patient reports and clinic meetings	Medicine<br>Nursing<br>Occupational therapy<br>Physiotherapy
Hallas, D., Fernandez, J. B., Herman, N. G., & Moursi, A. (2015). Identification of pediatric oral health core competencies through interprofessional education and	United States	Primary research<br>(Quantitative – nonexperimental descriptive study)	The purpose of this paper was to describe the design, implementation, and evaluation of dental and nursing interprofessional educational	*Primary care clinics*<br>- case presentation<br>- case studies	Dental students<br>Pediatric dental residents

**Author**	**Country**	**Literature Typology** (methodological approach, if applicable	**Purpose or Aim of Article**	***Setting*(s) IPP Education Approach/ Strategies**	**Student Disciplines Involved**
practice. *Nursing Research and Practice, 2015*, 360523.<br>DOI: 10.1155/2015/360523			activities designed to accomplish these goals.		Pediatric nurse practitioners
Hartnett, E., Haber, J., Catapano, P., Dougherty, N., Moursi, A. M., Kashani, R., Osman, C., Chinn, C., & Bella, A. (2019). The impact of an interprofessional pediatric oral health clerkship on advancing interprofessional education outcomes. *Journal of Dental Education, 83*(8), 878-886.<br>DOI: 10.21815/JDE.019.088	United States	Program evaluation<br>(Quantitative)	The aim of this study was to evaluate the effectiveness of an innovative pediatric interprofessional education clinical experience using oral-systemic health as the clinical population example for improving the self-reported interprofessional competencies of family nurse practitioner, dental, and medical students.	*Primary care clinics*<br>- clinical teams of students with trained pediatric dental residents<br>- pre-conference briefing<br>- collaboration in management plan and well-child visit<br>- post-conference debriefing	Dental students<br>Family nurse practitioner<br>Medical students
Hayward, K. S., Kochniuk, L., Powell, L., & Peterson, T. (2005). Changes in students' perceptions of interdisciplinary practice reaching the older adult through mobile service delivery. *Journal of Allied Health, 34*(4), 192-198.<br>DOI: no doi	United States	Primary research<br>(Quantitative – pretest/posttest design)	This article examined students' perceptions of interdisciplinary health care practice in a facilitated, community-based practicum experience.	*Rural community outreach*<br>- group work<br>- development of educational sessions	Dietetics<br>Health education<br>Nursing<br>Occupational therapy<br>Pharmacy<br>Physical therapy<br>Physician assistant<br>Social work

Author	Country	**Literature Typology** (methodological approach, if applicable	Purpose or Aim of Article	***Setting*(s)** **IPP Education Approach/ Strategies**	Student Disciplines Involved
Heath, J., Aker, R., Feld, H., Singer, R. L., & Norton, J. (2019). A pilot interprofessional program to promote oral health and wellness in Appalachian children. *Journal of Professional Nursing, 35*(5), 412-416. DOI: 10.1016/j.profnurs.2019.02.006	United States	Primary research (Quantitative – pretest/posttest design)	The aim of this article was to describe the outcomes from this innovative immersion experience to help bridge the gap with IPE and clinical practice in underserved communities through an academic-clinical partnership between seven health profession colleges.	*Elementary schools*<br>- direct care provision<br>- reflection	Dentistry<br>Health communication<br>Nursing<br>Pharmacy<br>Physical therapy<br>Public health<br>Social work
Hoover, M., & Andazola, J. (2012). Integrated behavioral care training in family practice residency: Opportunities and challenges. *Journal of Clinical Psychology in Medical Settings, 19*(4), 446-450. DOI: 10.1007/s10880-012-9353-9	United States	Program description	The paper described a family practice residency program that trains family medicine residents, prescribing psychology students, and pre-doctoral and post-doctoral behavioral health consultants.	*Health center*<br>- groupwork<br>- presentation	Counselling psychology doctoral students<br>Family medicine residents<br>Prescribing psychology practicum students
Hu, T., Cox, K. A., & Nyhof-Young, J. (2018). Investigating student perceptions at an interprofessional student-run free clinic serving marginalised populations. *Journal of Interprofessional Care, 32*(1), 75-79. DOI: 10.1080/13561820.2017.1363724	Canada	Primary research (Mixed methods)	The aim of this study was to evaluate the learning experiences and skills developed among interprofessional healthcare students at an SRFC serving marginalised populations, in order to provide data for ongoing programme improvement and recommendations for other SRFCs based on lessons learned.	*Community health centre*<br>- groupwork<br>- students from different disciplines paired for client interview and examination<br>- reflection session	Medicine<br>Nursing<br>Pharmacy<br>Physiotherapy<br>Social work

**Author**	**Country**	**Literature Typology** (methodological approach, if applicable	**Purpose or Aim of Article**	***Setting*(s) IPP Education Approach/ Strategies**	**Student Disciplines Involved**
Jackman, D., Yonge, O., Myrick, F., Janke, F., & Konkin, J. (2016). A rural interprofessional educational initiative: What success looks like. *Online Journal of Rural Nursing and Health Care, 16*(2), 5-26.<br>DOI: 10.14574/ojrnhc.v16i2.417	Canada	Primary research<br>(Qualitative – Glaserian grounded theory)	This article was an exploration of how participants defined a successful rural IPE experience, and which factors promoted or hindered that success.	*Rural community hospital*<br>- clinical placement<br>- clinical rounds	Medicine<br>Nursing
Jakobsen, F., Mørcke, A. M., Hansen, T. B. (2017). The interprofessional learning experience: Findings from a qualitative study based in an outpatient setting. *Journal of Interprofessional Care, 31*(5), 613-619.<br>DOI: 10.1080/13561820.2017.1322560	Denmark	Primary research<br>(Case study)	The purpose of this article was to enhance understanding of interprofessional learning during which students take care of authentic patients and acquire relevant learning experiences in an outpatient clinic setting with a pedagogical approach based on adult learning methods.	*Outpatient clinic*<br>- direct care provision<br>- reflective supervision	Medicine<br>Nursing
Jakobsen, F., Musaeus, P., Kirkeby, L., Hanse, T. B., & Mørcke, A. M. (2019). Emotions and clinical learning in an interprofessional outpatient clinic: a focused ethnographic study. *Journal of Interprofessional Care, 33*(1), 57-65.<br>DOI: 10.1080/13561820.2018.1514372	Denmark	Primary research<br>(Qualitative – ethnography)	The aim of this study was first to investigate the self-reported and the observed relationship of the four types of emotion: activity, outcome, epistemic and social emotions. Second to analyse the valence and activation of these types of emotions and their role in medical and nursing students' learning in a clinical interprofessional context.	*Outpatient clinic*<br>- direct care provision<br>- reflective supervision	Medicine<br>Nursing

Author	Country	**Literature Typology** (methodological approach, if applicable	Purpose or Aim of Article	***Setting*(s)** **IPP Education Approach/ Strategies**	Student Disciplines Involved
Janotha, B. L., Tamari, K., & Evangelidis-Sakellson, V. (2019). Dental and nurse practitioner student attitudes about collaboration before and after interprofessional clinical experiences. *Journal of Dental Education, 83*(6), 638-644.<br>DOI: 10.21815/JDE.019.073	United States	Primary research<br>(Quantitative – pre/post-test survey)	The aim of this study was to investigate the effect that an interprofessional clinical experience (IPCE) had on dental and adult-gerontology primary care nurse practitioner (NP) students' attitudes towards health care collaboration.	*Tertiary care center and dental clinic*<br>- direct care provision<br>- IPP groupwork	Dental student<br>Nurse practioner
Jentoft, R. (2021). Boundary-crossings among health students in interprofessional geropsychiatric outpatient practice: collaboration with elderly people living at home. *Journal of Interprofessional Care, 35*(3), 409-418.<br>DOI: 10.1080/13561820.2020.1733501	Norway	Primary research<br>(Qualitative – focused ethnography)	The purpose of this article was to understand how learning is shaped by interaction across boundaries in a new boundary-crossing practice.	*Outpatient mental health center*<br>- seminars<br>- direct care assessment	Medicine<br>Occupational therapy<br>Physical therapy
Johnson, K. L., Fuji, K. T., Franco, J. V., Castillo, S., O'Brien, K., & Begley, K. J. (2018). A pharmacist's role in a dental clinic: Establishing a collaborative and interprofessional education site. *Innovations in Pharmacy, 9*(4), 13.<br>DOI: 10.24926/iip.v9i4.1382	United States	Program description	The purpose of this paper was to describe the development of a formal interprofessional care team consisting of dentists, dental students, a pharmacist, and pharmacy students in a university dental school clinic.	*Dental clinic*<br>- direct care provision	Dentistry<br>Pharmacy
Jones, A. C., Li, T., Zomorodi, M., Broadhurst, R., & Weil, A. B. (2018). Straddling care and education:	United States	Program description	The purpose of this paper was to describe the experience with an an IP service-learning	*Hospital and community*	Chaplaincy<br>Medicine<br>Nursing

Author	Country	Literature Typology (methodological approach, if applicable	Purpose or Aim of Article	*Setting*(s) IPP Education Approach/ Strategies	Student Disciplines Involved
developing interprofessional collaboration through a hotspotting service learning project. *Healthcare, 6*(2), 108-109.<br>DOI: 10.1016/j.hjdsi.2018.02.001			hotspotting project, with also discussing the goals, interventions, achievement, challenges and perspective on scaling and sustaining these programs.	- direct care provision<br>- assessments	Pharmacy<br>Physician assistant<br>Public health<br>Social work<br>Undergraduate studies
Joseph, S., Diack, L., Garton, F., & Haxton, J. (2012). Interprofessional education in practice. *The Clinical Teacher, 9*(1), 27-31.<br>DOI: 10.1111/j.1743-498X.2011.00486.x	United Kingdom	Primary research<br>(Quantitative)	This article aimed to ascertain attitudinal change experienced by students undertaking IPE in clinical practice.	*Primary care centre*<br>- case observations<br>- case presentation	Medicine<br>Nursing<br>Occupational therapy<br>Pharmacy<br>Social work
Kent, F., Francis-Cracknell, A., McDonald, R., Newton, J. M., Keating, J. L., & Dodic, M. (2016). How do interprofessional student teams interact in a primary care clinic? A qualitative analysis using activity theory. *Advances in Health Sciences Education, 21*(4), 749-760.<br>DOI: 10.1007/s10459-015-9663-4	Australia	Primary research<br>(Qualitative – ethnography)	The aim of this paper was to investigate what and how students from differing professions learn together.	*Primary care and residential care*<br>- groupwork<br>- direct care provision<br>- case presentation<br>- reflection	Medicine<br>Nursing<br>Occupational therapy<br>Pharmacy<br>Physiotherapy
Kipp, J., Pimlott, J. F., & Satzinger, F. (2007). Universities preparing health professionals for the 21st century: can something new come out of the traditional establishment? *Journal of*	Canada	Program evaluation<br>(Mixed methods)	An aim/purpose of article was not explicity stated. Authors provided a description and background for an Enhancing Clinical Capacity Project Fund	*Unclear*<br>- interdisciplinary team placements	Medicine<br>Nursing<br>Nutrition<br>Pharmacy

**Author**	**Country**	**Literature Typology** (methodological approach, if applicable	**Purpose or Aim of Article**	***Setting*(s)** **IPP Education Approach/ Strategies**	**Student Disciplines Involved**
Interprofessional Care, 21(6), 633-644.<br>DOI: 10.1080/13561820701697844			project that established student team placements within a chronic disease model for diabetic services.		
Kostoff, M. D., & Shin, T. R. (2016). Integration of pharmacy students into family medicine residency clinics. *Family Medicine, 48*(10), 805-808.<br>DOI: no doi	United States	Primary research<br>(Mixed methods)	The purpose of this study was to determine the impact of pharmacy student integration into a family medicine residency clinic on family medicine residents' attitudes toward interprofessional collaboration and satisfaction.	*Primary care clinic*<br>- direct care provision<br>- groupwork	Family medicine residents<br>Pharmacy
Kuder, L. C., Gairola, G. A., & Hamilton, C. C. (2001). Development of rural interdisciplinary geriatrics teams. *Gerontology & Geriatrics Education, 21*(4), 65-79.<br>DOI: 10.1300/J021v21n04_06	United States	Program description	This article described a project to develop interdisciplinary geriatrics teams and student training sites in rural Appalachian primary care settings.	*Primary care clinic*<br>- direct care provision<br>- assessments<br>- case conferences	Allied health<br>Dentistry<br>Medicine<br>Nursing<br>Pharmacy<br>Social work
Lai, C. J., Nye, H. E., Bookwalter, T., Kwan, A., & Hauer, K. E. (2008). Postdischarge follow-up visits for medical and pharmacy students on an inpatient medicine clerkship. *Journal of Hospital Medicine, 3*(1), 20-27.<br>DOI: 10.1002/jhm.264	United States	Primary research<br>(Quantitative – pre/post survey)	The purpose of the study was to assess the impact of this didactic and experiential curriculum on students' attitudes and self-assessed skills in the interdisciplinary care and transitional care of chronically ill patients.	*Tertiary university hospital*<br>- groupwork<br>- care provision<br>- debriefing session	Medicine<br>Pharmacy

Author	Country	Literature Typology (methodological approach, if applicable	Purpose or Aim of Article	*Setting*(s) IPP Education Approach/ Strategies	Student Disciplines Involved
Lauckner, H. M., Nickerson Rak, C., Hickey, E. M., Isenor, J. E., & Godden-Webster, A. L. (2018). Interprofessional and collaborative care planning activities for students and staff within an academic nursing home. *Journal of Interprofessional Education & Practice, 13*, 1-4. DOI: 10.1016/j.xjep.2018.07.005	Canada	Primary research (Quantitative – not specified)	An aim/purpose of article was not explicity stated. Authors described interprofessional team experiences in an academic nursing home, and discussed the IPE curriculum and student outcome.	*Academic nursing home*<br>- weekly facilitated meetings to coduct assessments and develop care plans<br>- shadowing providers<br>- participate in team meetings	Dietetics<br>Nurse practitioners<br>Occupational therapy<br>Pharmacy<br>Speech-language pathology<br>Social Work<br>Therapeutic recreation
Lidskog, M., Löfmark, A., & Ahlström, G. (2009). Learning through participating on an interprofessional training ward. *Journal of Interprofessional Care, 23*(5), 486-497. DOI: 10.1080/13561820902921878	Sweden	Primary research (Case study)	The aim of this study was to describe and analyse the students' learning on an interprofessional training ward within care for older persons through focusing on the students' ways of participating in the community of practice on the ward. Learning through participating on a training ward.	*Nursing home*<br>- groupwork in IPP teams<br>- direct care provision	Nursing<br>Occupational therapy<br>Social work
Mann, K. V., Mcfetridge-Durdle, J., Martin-Misener, R., Clovis, J., Rowe, R., Beanlands, H., & Sarria, M. (2009). Interprofessional education for students of the health professions: The "Seamless Care" model. *Journal*	Canada	Program description	This paper aimed to describe the Seamless Care model of interprofessional education and discusses the theoretical underpinnings of this experiential model of	*Quaternary-care hospital*<br>- IPP groupwork<br>- development of patient care plan	Dental hygiene<br>Dentistry<br>Medicine<br>Nursing<br>Pharmacy

Author	Country	Literature Typology (methodological approach, if applicable	Purpose or Aim of Article	*Setting*(s) IPP Education Approach/ Strategies	Student Disciplines Involved
of Interprofessional Care, 23(3), 224-233.<br>DOI: 10.1080/13561820802697735			interprofessional education deisnged to extend classroom-based interprofessional education to the clinical setting.	- interprofessional modules<br>- reflective exercise	
Mattison, D., Smith, L. J., Balzer, K., Bavireddy, V., Bishop, T. W., Farris, K., Fitzgerald, M., Rulli, D., Trupiano, N., & Anderson, O. S. (2023). Longitudinal Interprofessional Family Based Experience (LIFE): An authentic experiential interprofessional education learning framework. *Pedagogy in Health Promotion, 9*(1), 45-53.<br>DOI: 10.1177/23733799211060726	United States	Program evaluation<br>(Quantitative)	This paper introduced the conceptual framework of an unique IPE experiential opportunity that moved beyond a one time intervention and use of cases or simulated patients, to a longitudinal experience with innovative collaborations that engage real patients and families in the learning process.	*Community-based virtual visits (Zoom)*<br>- pre-work and kick-off event<br>team visits with patient/family through Zoom<br>- team debriefings after patient-family meetings<br>- wrap-up session with reflection on experience	Dentistry<br>Health science<br>Kinesiology<br>Public health<br>Social work
McCombe, J., Develin, D., & Mallik, M. (2008). Creating interprofessional learning capacity in children's centres – description and evaluation of a pilot project. *Learning in Health and Social Care, 7*(4), 235-246.<br>DOI: 10.1111/j.1473-6861.2008.00199.x	United Kingdom	Program description	This article described the setting up, implementation and evaluation of a pilot project to ensure a focus on interprofessional learning for all undergraduate/pre-registration students of health and social care accessing placement learning in children's centres.	*Community children center*<br>- workshops	Nursing<br>Social work

**Author**	**Country**	**Literature Typology** (methodological approach, if applicable	**Purpose or Aim of Article**	***Setting*(s)** **IPP Education Approach/ Strategies**	**Student Disciplines Involved**
McElfish, P. A., Moore, R., Buron, B., Hudson, J., Long, C. R., Purvis, R. S., Schulz, T. K., Rowland, B., & Warmack, T. S. (2018). Integrating interprofessional education and cultural competency training to address health disparities. *Teaching and Learning in Medicine, 30*(2), 213-222.<br>DOI: 10.1080/10401334.2017.1365717	United States	Primary research<br>(Mixed methods)	This article presented an evaluation of an innovative pilot program that combined IPE and cultural competency learning opportunities through (a) educational seminars, (b) clinical experiential learning in a studentled clinic, and (c) community-based service-learning through health assessments and survey research events.	*Student-led clinic*<br>- educational seminars<br>- clinical experiential learning in student-led clinic<br>- community-based service learning<br>- direct care provision	Medicine<br>Nursing<br>Pharmacy<br>Radiologic imaging science
McNair, R., Brown, R., Stone, N., & Sims, J. (2001). Rural interprofessional education: promoting teamwork in primary health care education and practice. *The Australian Journal of Rural Health, 9*(Suppl 1), S19-S26.<br>DOI: 10.1046/j.1440-1584.9.s1.9.x	Australia	Program description	The present paper describes the context for an evolving interprofessional education project in rural primary care, designed to promote collaboration.	*Rural community health center*<br>- community engagement<br>- case presentation<br>- structured debriefing<br>- reflective discussion	Medicine<br>Nursing
McNair, R., Stone, N., Sims, J., & Curtis, C. (2005). Australian evidence for interprofessional education contributing to effective teamwork preparation and interest in rural practice. *Journal of Interprofessional Care, 19*(6), 579-594.	Australia	Primary research<br>(Quantitative – before/after quasi-experimental)	This paper outlined the educational model, of a pilot placement for undergraduate health care professional students in rural Victoria, Australia, then focuses on the evaluation	*Rural community health center*<br>- clinical placement<br>- discussion forum<br>- action-based learning	Medicine<br>Nursing<br>Pharmacy<br>Physiotherapy

Author	Country	Literature Typology (methodological approach, if applicable	Purpose or Aim of Article	*Setting*(s) IPP Education Approach/ Strategies	Student Disciplines Involved
DOI: 10.1080/13561820500412452			methods and results obtained from student evaluations.	- case presentation	
Mecca, M. C., Thomas, J. M., Niehoff, K. M., Hyson, A., Jeffery, S. M., Sellinger, J., Mecca, A. P., Van Ness, P. H., Fried, T. R., & Brienza, R. (2019). Assessing an interprofessional polypharmacy and deprescribing educational intervention for primary care post-graduate trainees: a quantitative and qualitative evaluation. *Journal of General Internal Medicine*, *34*(7), 1220-1227.<br>DOI: 10.1007/s11606-019-04932-9	United States	Primary research<br>(Mixed methods – exploratory, sequential design)	This article aimed to assess residents' knowledge of polypharmacy and perceptions of this interprofessional education intervention.	*Primary care*<br>- case conference<br>- direct care provision	Internal medicine<br>Nurse practitioner
Mihalynuk, T. V., Soule Odegarrd, P., Kang, R., Kedzierski, M., & Crowley, N. J. (2007). Partnering to enhance interprofessional service-learning innovations and addictions recovery. *Education for Health, 20*(3), 92.<br>DOI: no doi	United Staes	Program description	This article described the creation and delivery of University of Washington- Adult Rehabilitation Center interprofessional service-learning innovations to enhance student learning and addictions recovery.	*Adult rehabilitation center*<br>- weekly 3-hour on-site experience<br>- opportunities to work with ARC staff<br>- development of education modules and materials	Dentistry<br>Information sciences<br>Medicine<br>Nursing<br>Public health and community medicine<br>Social work
Miselis, H. H., Zawacki, S., White, S., Yinusa-Nyahkoon, L., Mostow, C., Furlong, J., Mott, K. K., Kumar, A.,	United States	Primary research<br>(Mixed methods)	The goal of this paper was to report on a study of the impact of IPE in longitudinal priary care	*Family medicine clinics*	Medicine<br>Physician assistant

Author	Country	Literature Typology (methodological approach, if applicable	Purpose or Aim of Article	*Setting*(s) IPP Education Approach/ Strategies	Student Disciplines Involved
Winter, M. R., Berklein, F., & Jack, B. (2022). Interprofessional education in the clinical learning environment: a mixed-methods evaluation of a longitudinal experience in the primary care setting. *Journal of Interprofessional Care, 36*(6), 845-855. DOI: 10.1080/13561820.2022.2025768			practices on trainees' interprofessional knowledge, attitudes, skills, and behaviors.	- case-based didactic discussions<br>- team huddles<br>- patient visits<br>- debriefings	Nutrition and dietetics<br>Social work
Moreno-Vasquez, A., Gandara, E., Idar, A. Z., Recto, P., Zapata, J., & Lesser, J. (2021). Developing and implementing a co-curricular IPE program: AHEC scholars program. *Public Health Nursing, 38*(6), 1080-1087. DOI: 10.1111/phn.12947	United States	Primary research (Mixed methods – pre/post quasi-experimental; reflective journaling)	The purpose of this article is twofold: firstly, to present the process of the development and implementation of the AHEC Scholars Program; secondly, to present the outcomes from a 3-month pilot study.	*Federally qualified health center*<br>- didactic work<br>- direct care provision	Dentistry<br>Healthcare administration<br>Medicine<br>Nursing<br>Pharmacy<br>Public health<br>(mentions other unspecified health professions)
Mpofu, R., Daniels, P. S., Adonis, T. A., & Karuguti, W. M. (2014). Impact of an interprofessional education program on developing skilled graduates well-equipped to practise in rural and underserved areas. *Rural and Remote Health, 14*(3), 2671.	South Africa	Primary research (Mixed methods – questionnaire; focus groups)	This article reports on an investigation of the perceptions of students in an IPE program experience, and how their perceptions of this experience influenced their intention for	*Rural and underserved community*<br>- groupwork<br>- structured reflection	Natural medicine<br>Nursing<br>Physiotherapy

Author	Country	Literature Typology (methodological approach, if applicable	Purpose or Aim of Article	*Setting*(s) IPP Education Approach/ Strategies	Student Disciplines Involved
DOI: 10.22605/RRH2671			future practice within rural South African communities.	- case presentation	
Müller, J., & Couper, I. (2021). Preparing graduates for interprofessional practice in South Africa: The dissonance between learning and practice. *Frontiers in Public Health, 9*, 594894.<br>DOI: 10.3389/fpubh.2021.594894	South Africa	Primary research<br>(Qualitative – interviews)	This paper reports on an explorative pilot study investigating students' and graduates' perceived value of their interprofessional home visit exposure in preparing them for working in South Africa.	*Home visits*<br>- direct care provision<br>- reflection session	Human nutrition<br>Medicine<br>Occupational therapy<br>Physiotherapy<br>Speech language and hearing therapy
Nagelkerk, J., Thompson, M. E., Bouthillier, M., Tompkins, A., Baer, L. J., Trytko, J., Booth, A., Stevens, A., & Groeneveld, K. (2018). Improving outcomes in adults with diabetes through an interprofessional collaborative practice program. *Journal of Interprofessional Care, 32*(1), 4-13.<br>DOI: 10.1080/13561820.2017.1372395	United States	Primary research<br>(Mixed methods – sequential design, pre-post questionnaires, focus groups)	The purpose of the research was to test the implementation of an IPCP program in a family practice FQHC that included interprofessional teams of students in the provision of care to determine if IPCP activities and student team members improved patient outcomes and staff productivity.	*Federally qualified health center*<br>- IP groupwork<br>- direct care provision<br>- student-led classes<br>- daily huddles	Medicine<br>Pharmacy<br>Physician assistant
Nagelkerk, J., Trytko, J., Baer, L. J., Tompkins, A., Thompson, M., Bouthillier, M., Booth, A., & Nord, C. (2021). Sustainability of an IPCP program within a federally qualified health center including interprofessional student team	United States	Primary research<br>(Mixed methods – longitudinal design)	The purpose of this article is to describe the continued implementation of The Midwest Interprofessional Practice, Education, and Research Center (MIPERC) IPCP program at CSHS, a family practice clinic	*Federally qualified health center*<br>- IP groupwork<br>- direct care provision<br>- student-led classes	Medical assistant<br>Medicine<br>Nurse practitioner<br>Pharmacy<br>Physician assistant<br>Social work

Author	Country	Literature Typology (methodological approach, if applicable	Purpose or Aim of Article	*Setting*(s) IPP Education Approach/ Strategies	Student Disciplines Involved
placements. *Journal of Interprofessional Care, 35*(6), 869-877.<br>DOI: 10.1080/13561820.2020.1816935			within a Federally Qualified Health Center (FQHC), located in Grand Rapids, Michigan, and changes to the composition of interdisciplinary teams of students over two years.	- daily huddles	
Norbye, B. (2016). Healthcare students as innovative partners in the development of future healthcare services: An action research approach. *Nurse Education Today, 46*, 4-9.<br>DOI: 10.1016/j.nedt.2016.06.021	Norway	Primary research<br>(Qualitative – action research, interviews, field notes, reflection logs)	This study aimed to explore how partnerships for the contextualisation of learning environments in community health services can contribute to the knowledge and requirements of new methods of working and also presents unintentional outcomes of interprofessional learning when student teams worked with patients selected by healthcare services.	*Rural health services*<br>- IP groupwork<br>- direct care provision<br>- daily meetings	Medicine<br>Nursing<br>Occupational therapy<br>Physiotherapy
Normandin, C., Hayes, V., Cyr, P., & Schirmer, J. (2023). Implementation and impact of an interprofessional education curriculum on medical, pharmacy, and social work students' attitudes, perceptions, and self-assessed teamwork skills. *Journal of Interprofessional Care, 37*(5), 846-850.<br>DOI: 10.1080/13561820.2023.2169260	United States	Primary research<br>(Quantitative)	An aim/purpose of article was not explicity stated. Authors described the implementation and assessment of impact of a practice-based IPE curriculum involving direct patient care on student perceptions of competency and autonomy within their own profession, on attitudes toward the contributions and value of other	*Residency training site, inpatient service, and a family medicine clinic*<br>- bi-weekly IP home visits<br>- collaborative progress notes and 30-minute IPE	Medicine<br>Pharmacy<br>Social work

**Author**	**Country**	**Literature Typology** (methodological approach, if applicable	**Purpose or Aim of Article**	***Setting*(s)** **IPP Education Approach/ Strategies**	**Student Disciplines Involved**
			professions and individual self-assessment of teamwork skills.	discussion with faculty<br>- weekly IP interview of patient<br>- pre-interview huddle/post-intervention debrief	
O'Brien, D., Swann, J., & Heap, N. (2015). Can the communities of practice model explain the complex organization of an interprofessional student-led health clinic? *Journal of Allied Health, 44*(1), e11-e16.<br>DOI: no doi	New Zealand	Program description	This paper outlined the organization of one such clinic (student-led university-based health clinic), and provides an insight to the processes by which IPCP and IPE are achieved in this setting. It explores the clinic using the CoP model as a framework for analysis.	*Community clinic*<br>- weekly in-service sessions<br>- IPE tutorials<br>- case conferences	Counselling psychology<br>Nursing<br>Occupational therapy<br>Oral health<br>Physical therapy<br>Podiatry<br>Psychotherapy<br>Speech and language therapy
O'Carroll, V., Smith, J., Collinson, A., Jackson, C., & Ker, J. (2013). Interprofessional education for students' in primary care placements: Widening opportunities for accessible interprofessional learning. *Medical Science Educator, 23*(3), 405-410.<br>DOI: 10.1007/BF03341656	Scotland	Program description	This paper described an interprofessional activity which enables students to experience interprofessional learning and working within the primary care setting and to consider the impact of effective teamwork on patient safety.	*Home visits through primary care practices*<br>- worked paired in small teams<br>- planned and carried out home visits	Medicine<br>Nursing

**Author**	**Country**	**Literature Typology** (methodological approach, if applicable	**Purpose or Aim of Article**	***Setting*(s) IPP Education Approach/ Strategies**	**Student Disciplines Involved**
				- meetings with IPE coordinator and mentor for reflection and discussion<br>- optional reflective piece	
Palmer, R. T., & Stilp, C. (2017). Learning by doing: The MD-PA interprofessional education rural rotation. *Rural and Remote Health, 17*(1), 4167.<br>DOI: 10.22605/rrh4167	United States	Program description	An aim/purpose of article was not explicity stated. Authors provided a detailed description of the Combined Medical-Physician Assistant Student Rural Rotation program.	*Rural community engagement*<br>- community project<br>- goal identification and reflection<br>- case presentation	Medicine<br>Physician assistant
Philippon, D. J., Pimlott, J. F., King, S., Day, R. A., & Cox, C. (2005). Preparing health science students to be effective health care team members: the interprofessional initiative at the university of Alberta. *Journal of Interprofessional Care*, *19*(3), 195-206.<br>DOI: 10.1080/13561820500126839	Canada	Program description	This paper traced the evolution of the Interprofessional Initiative at the University of Alberta over the past decade and describes future directions.	*Specialty clinic and tertiary care center*<br>- IP groupwork<br>- case conferences	Dentistry<br>Home economics<br>Kinesiology<br>Medical laboratory science<br>Medicine<br>Nursing<br>Nutrition & dietetics<br>Occupational therapy<br>Pharmacy<br>Physical education

Author	Country	Literature Typology (methodological approach, if applicable	Purpose or Aim of Article	*Setting*(s) IPP Education Approach/ Strategies	Student Disciplines Involved
					Physical therapy<br>Recreation & leisure studies
Pippitt, K., Moloney-Johns, A., Jalilibahabadi, S., & Gren, L. H. (2015). Collaboration versus competition: An interprofessional education experience. *Family medicine*, *47*(4), 298-301.<br>DOI: no doi	United States	Program evaluation<br>(Qualitative)	The purpose of this article was to assess student, preceptor, and patient satisfaction with a phased pilot project to introduce interprofessional education teams into a clinical setting.	*Outpatient clinics*<br>- IP groupwork<br>- direct care provision<br>- debriefings	Medicine<br>Physician assistant
Porcerelli, J. H., Fowler, S. L., Murdoch, W., Markova, T., & Kimbrough, C. (2013). Training family medicine residents to practice collaboratively with psychology trainees. *The International Journal of Psychiatry in Medicine*, *45*(4), 357-365.<br>DOI: 10.2190/PM.45.4.f	United States	Program description	The purpose of this article was to describe a training curriculum for family medicine residents to practice collaboratively with psychology (doctoral) trainees at the Wayne State University/Crittenton Family Medicine Residency program.	*Outpatient clinic*<br>- huddle<br>- shadowing<br>- co-counselling<br>- shared precepting<br>- lectures<br>- video-observation and feedback<br>- direct care provision<br>- projects	Medical residents<br>Psychology trainees
Powell, K., Stocks, N., & Laurence, C. (2016). A new venture in interdisciplinary student learning in a co-located health service. *Australian Health Review*, *40*(2), 205-209.	Australia	Primary research<br>(Case study)	The purpose of this case study was to describe how the standard student placement experience ('shadowing' a practitioner) may be enhanced with a	*Primary care clinic*<br>- case conferences<br>- informal discussions	Counselling<br>Exercise physiology<br>Medicine

Author	Country	Literature Typology (methodological approach, if applicable	Purpose or Aim of Article	*Setting*(s) IPP Education Approach/ Strategies	Student Disciplines Involved
DOI: 10.1071/AH14241			interdisciplinary program in a health service clinic.		Nursing<br>Physiotherapy<br>Psychology
Rose, M. A., Lyons, K. J., Miller, P. K. S., & Cornman-Levy, D. (2003). The effect of an interdisciplinary community health project on student attitudes toward community health, people who are indigent and homeless, and team leadership skill development. *Journal of allied health*, *32*(2), 122-125.<br>DOI: no doi	United States	Primary research<br>(Quantitative)	The purpose of this article was to examine whteher students' attitudes about community health practice, attitudes toward people who are indigent and homeless, and perceived leadership skills changed after participation in a planned interdisciplinary community health experience with an urban homeless or formerly homeless population. It was also to describe an interdisciplinary community health project, based on the Community Health Empowerment Model (CHEM), for graduate students in nursing, medicine, occupational therapy, physical therapy and social work.	*Homeless shelters and transitional housing facilities*<br>- shared practicum experience	Medicine<br>Nursing<br>Occupational therapy<br>Physical therapy<br>Social work
Ryan, M., Vanderbilt, A. A., Mayer, S. D., & Gregory, A. (2015). Interprofessional education as a method to address health needs in a Hispanic community setting: A pilot	United States	Primary research<br>(Quantitative – pre/post test design)	An aim/purpose of article was not explicity stated. Authors compared student attitudes towards community service before and after their experience.	*Collaboration between community resources and university*	Medicine<br>Nursing<br>Pharmacy

Author	Country	Literature Typology (methodological approach, if applicable	Purpose or Aim of Article	*Setting*(s) IPP Education Approach/ Strategies	Student Disciplines Involved
study. *Journal of Interprofessional Care*, *29*(5), 515-517. DOI: 10.3109/13561820.2015.1020360				- direct care provision	
Saunders, R., Dugmore, H., Seaman, K., Singer, R., & Lake, F. (2019). Interprofessional learning in ambulatory care. *The Clinical Teacher*, *16*(1), 41-46. DOI: 10.1111/tct.12764	Australia	Program evaluation (Mixed methods)	The purpose of this study was to evaluate an interprofessional placement in ambulatory care for medical and nursing students.	*Specialty clinics*<br>- IP groupwork<br>- case conferences<br>- direct care provision	Medicine<br>Nursing
Schellack, N., Wium, A. M., Ehlert, K., van Aswegen, Y., & Gous, A. (2015). Establishing a pharmacotherapy induced ototoxicity programme within a service-learning approach. *South African Journal of Communication Disorders*, *62*(1), 1-7. DOI: 10.4102/sajcd.v62i1.96	South Africa	Program description	The main aim of this article was to describe the collaboration between two disciplines (SMU – Department of Pharmacy and the Discipline of Speech-Language Pathology and Audiology) in the initiation of a pharmacotherapy induced ototoxicity clinic within a servicelearning approach	*Tertiary hospital*<br>- service-learning activity<br>- IP team meetings<br>- reflective journals	Audiology<br>Pharmacy
Seaman, K., Saunders, R., Dugmore, H., Tobin, C., Singer, R., & Lake, F. (2018). Shifts in nursing and medical students' attitudes, beliefs and behaviours about interprofessional work: An interprofessional placement in ambulatory care. *Journal of Clinical Nursing*, *27*(15-16), 3123-3130.	Australia	Primary research (Mixed methods)	The purpose of this article was to examine students' beliefs, behaviours and attitudes in relation to interprofessional socialisation, and their expectations and experience, before and after a 2-week clinical placement in ambulatory care.	*Outpatient clinics*<br>- IP groupwork<br>- direct care provision	Medicine<br>Nursing

**Author**	**Country**	**Literature Typology** (methodological approach, if applicable	**Purpose or Aim of Article**	***Setting*(s) IPP Education Approach/ Strategies**	**Student Disciplines Involved**
DOI: 10.1111/jocn.14506					
Seif, G., Coker-Bolt, P., Kraft, S., Gonsalves, W., Simpson, K., & Johnson, E. (2014). The development of clinical reasoning and interprofessional behaviors: service-learning at a student-run free clinic. *Journal of Interprofessional Care*, *28*(6), 559-564. DOI: 10.3109/13561820.2014.921899	United States	Primary research (Quantitative – prospective cohort quasi-experimental pre/post test design)	The purpose of this article was to examine the benefits of utilizing SRFCs as a service learning experience that not only improves access to patient care but also fosters positive interprofessional attitudes and enhances students' clinical reasoning skills.	*Student-run clinic*<br>- lecture<br>- clinical skills practice<br>- direct care provision	Medicine<br>Occupational therapy<br>Pharmacy<br>Physical therapy<br>Physician assistant
Shakoor, K., Boelens, L., & Yingling, C. (2023). Improving nurse practitioner practice readiness in an interprofessional student-run free clinic. *Journal for Nurse Practitioners, 19*(7), 104647. DOI: 10.1016/j.nurpra.2023.104647	United States	Program description	This report described the process and benefits of shared learning experiences between students in various health programs.	*Student-run free clinic (rural)*<br>- - team-based care delivery<br>- joint patient history taking<br>- shared workspaces to facilitate collaboration	Medicine<br>Nursing<br>Nurse practitioner<br>Pharmacy<br>Public health<br>Social work
Sicat, B. L., Huynh, C., Willett, R., Polich, S., & Mayer, S. (2014). Interprofessional education in a primary care teaching clinic: findings from a study involving pharmacy and medical students. *Journal of Interprofessional Care*, *28*(1), 71-73. DOI: 10.3109/13561820.2013.829424	United States	Primary research (Quantitative – pre/post surveys)	The purpose of this study was to examine the impact of that IPE experience on students' perceptions and attitudes towards one another and towards team-based patient care.	*Primary care clinic*<br>- patient interview<br>- case conference	Medicine<br>Pharmacy

Author	Country	**Literature Typology** (methodological approach, if applicable	Purpose or Aim of Article	***Setting*(s)** **IPP Education Approach/ Strategies**	Student Disciplines Involved
Snyman, S., & Donald, H. (2019). Interprofessional service-learning: Cutting teeth and learning to crawl. *Journal of Interprofessional Care*, *33*(3), 328-335.<br>DOI: 10.1080/13561820.2019.1611288	South Africa	Primary research<br>(Qualitative – interpretive qualitative approach)	The study aimed to determine how this learning experience influenced students' interprofessional person-centered practice.	*Community based organizations*<br>- community needs assessments<br>- planning and development of intervention<br>- implementation of plan and working as "case managers"	Dietetics<br>Medicine<br>Occupational therapy
Solomon, P., & Jung, B. (2006). An interprofessional role-emerging placement in HIV rehabilitation. *International Journal of Therapy and Rehabilitation*, *13*(2), 59-65.<br>DOI: 10.12968/ijtr.2006.13.2.21354	Canada	Program description	The purpose of this article was to highlight the current issues related to OT and PT management of people living with HIV, to describe the development and evaluation of a role-emerging, IP clinical placement at a community health centre servicing patients with HIV and to discuss the learning points from students' perspectives.	*Community health centre*<br>- needs assessment<br>- case planning and development<br>- case presentation	Occupational therapy<br>Physical therapy
Street, K. N., Eaton, N., Clarke, B., Ellis, M., Young, P. M., Hunt, L., & Emond, A. (2007). Child disability case studies: An interprofessional learning opportunity for medical students and paediatric nursing	United Kingdom	Primary research<br>(Mixed methods – randomized control study)	An aim/purpose of article was not explicity stated but the authors describe the development and evaluation of a practice-based interprofessional learning opportunity for pre-	*Home and school visits*<br>- worked in IP pairs<br>- home and school visits with a child	Medicine<br>Nursing

Author	Country	Literature Typology (methodological approach, if applicable	Purpose or Aim of Article	*Setting*(s) IPP Education Approach/ Strategies	Student Disciplines Involved
students. *Medical Education, 41*(8), 771-780.<br>DOI: 10.1111/j.1365-2923.2007.02800.x			qualification medical and pediatric nursing students using community-based studies of children with disabilities and their family.	- presentation to peers with feedback from tutor	
Tervaskanto-Maentausta, T., Taanila, A., Ukkola, O., Mikkila, L., Jokinen, J., & Varkki, E. (2017). Collaborative diabetes training in outpatient primary care. *Journal of European CME, 6*(1), 1288490.<br>DOI: 10.1080/21614083.2017.1288490	Finland	Primary research<br>(Quantitative)	This paper described the development and evaluation of a practice-based IPL opportunity.	*Primary care and specialized hospital settings*<br>- IP triads planned and conducted outpatient client visits<br>- joint discussions with facilitating physician and nurse<br>- reflective debriefing	Medicine<br>Nursing
Toth-Pal, E., Fridén, C., Asenjo, S. T., & Olsson, C. B. (2020). Home visits as an interprofessional learning activity for students in primary healthcare. *Primary Health Care Research & Development*, *21*, e59.<br>DOI: 10.1017/S1463423620000572	Sweden	Primary research<br>(Mixed methods – likert scale, focus groups)	The aim of this project was to implement and evaluate person-centred home visits as an educational model of IPL in primary healthcare during healthcare students' clinical practice.	*Primary healthcare units*<br>- direct care provision<br>- care planning<br>- case conference<br>- reflections	Dietitian<br>Medicine<br>Nursing<br>Occupational therapy<br>Physical therapy<br>Speech therapy
van Lierop, M., van Dongen, J., Janssen, M., Smeets, H., van Bokhoven, L., &	Netherlands	Program description	The purpose of this article was to describe a program where the	*Primary care clinic*	Medicine<br>Nursing

**Author**	**Country**	**Literature Typology** (methodological approach, if applicable	**Purpose or Aim of Article**	***Setting*(s) IPP Education Approach/ Strategies**	**Student Disciplines Involved**
Moser, A. (2019). Jointly discussing care plans for real-life patients: the potential of a student-led interprofessional team meeting in undergraduate health professions education. *Perspectives on Medical Education*, *8*, 372-377.<br>DOI: 10.1007/s40037-019-00543-6			authors developed a learning activity in which undergraduate medical, nursing and allied healthcare students practice interprofessional collabpration during a student-led interprofessional team meeting.	- direct care provision<br>- care planning and review<br>- case conferences<br>- team reflection	Occupational therapy<br>Physiotherapy<br>Speech and language therapy
Wang, J., Hu, X., Liu, J., & Li, L. (2016). Pharmacy students' attitudes towards physician-pharmacist collaboration: Intervention effect of integrating cooperative learning into an interprofessional team-based community service. *Journal of Interprofessional Care, 30*(5), 591-598.<br>DOI: 10.1080/13561820.2016.1185095	China	Primary research<br>(Quantitative - Quasi-experimental pre/post intervention)	The aim of this study was to evaluate the attitudes towards physician-pharmacist collaboration among pharmacy students in order to develop an IPE opportunity through integrating cooperative learning into team-based student-supported community service event.	*Local community settings*<br>- initial meeting to bring students together for IPE activity and organize teams<br>- cooperative learning tasks assigned to teams<br>- group reports on preparatory work<br>- negotiation of roles and responsibilities<br>- discipline-specific assessments and education	Medicine<br>Pharmacy
Wang, T., & Bhakta, H. (2013). A new model for interprofessional collaboration at a student-run free	United States	Program description	An aim/purpose of article was not explicity stated. Authors	*Student-run clinic*	Graduate nursing<br>Medicine

Author	Country	Literature Typology (methodological approach, if applicable	Purpose or Aim of Article	*Setting*(s) IPP Education Approach/ Strategies	Student Disciplines Involved
clinic. *Journal of Interprofessional Care*, *27*(4), 339-340.<br>DOI: 10.3109/13561820.2012.761598			provided a description of the program.	- direct care provision<br>- case presentation	
Warner, P., Jelinek, H., & Davidson, P. M. (2010). A university clinic: an innovative model for improving clinical practice. *Australian Journal of Advanced Nursing, The*, *27*(4), 38-42.<br>DOI: no doi	Australia	Primary research<br>(Quantitative)	An aim/purpose of article was not explicity stated. Authors appeared to evaluate an interdisciplinary program designed to improve and extend clinical skills of undergraduate health care students.	*University campus*<br>- clinical skills station<br>- IP groupwork sharing/exchanging discipline specific knowledge<br>- debriefing sessions	Undergraduate nursing<br>Undergraduate podiatry
Weinstein, A. R., Dolce, M. C., Koster, M., Parikh, R., Hamlyn, E., McNamara, E. A., Carlson, A., & DiVall, M. V. (2018). Integration of systematic clinical interprofessional training in a student-faculty collaborative primary care practice. *Journal of Interprofessional Care*, *32*(1), 104-107.<br>DOI: 10.1080/13561820.2017.1355296	United States	Primary research<br>(Quantitative – pre/post intervention design)	An aim/purpose of article was not explicity stated. Authors presented an evaluation of the perceived impact of IPE curriculum/practice on students' attitude towards team-based care.	*Tertiary medical center*<br>- IP groupwork<br>- huddles<br>- IP direct care provision<br>- debrief session	Masters of public health<br>Medicine<br>Nurse practitioner<br>Pharmacy
Weller-Newton, J. M., & Kent, F. (2021). Community health placements for junior medical and nursing students for interprofessional learning. *Journal of Interprofessional Care*, *35*(2), 316-319.	Australia	Primary research<br>(Mixed methods)	The purpose of this article was to investigate the value of interprofessional clinical placements at a community health center for dyads of second	*Community health services*<br>- consultation observation	Medicine<br>Nursing

**Author**	**Country**	**Literature Typology** (methodological approach, if applicable	**Purpose or Aim of Article**	***Setting*(s) IPP Education Approach/ Strategies**	**Student Disciplines Involved**
DOI: 10.1080/13561820.2020.1760803			year medical and nursing students.		
Weppner, W. G., Davis, K., Sordahl, J., Willis, J., Fisher, A., Brotman, A., Tivis, R., Gordon, T., & Smith, C. S. (2016). Interprofessional care conferences for high-risk primary care patients. *Academic Medicine*, *91*(6), 798-802.<br>DOI: 10.1097/ACM.0000000000001151	United States	Program description	An aim/purpose of article was not explicity stated. Authors described a program design and implementation of the PACT-ICU (Patient-aligned Care Team Interprofessional Care Update)	*Veteran affairs center*<br>- case presentation<br>- conference sessions<br>- clinical training	Clinical psychology<br>Nurse practitioners<br>Nursing<br>Pharmacy<br>Physician residents<br>Social work
